# AI-Driven Workflow for Automated Colorimetric Isothermal Amplification for Robust Pathogen Detection on Portable Devices

**DOI:** 10.1021/acsomega.5c12299

**Published:** 2026-08-05

**Authors:** Ke Xu, Matthew L. Cavuto, Kenny Malpartida-Cardenas, Marcus Pond, Dimbintsoa Rakotomalala Robinson, Francois Kiemde, Bernard Hernandez, Shiranee Sriskandan, Umberto D’Alessandro, Annette Erhart, Halidou Tinto, Aubrey J. Cunnington, Alison Holmes, Pantelis Georgiou, Nicolas Moser, Jesus Rodriguez-Manzano

**Affiliations:** † Department of Infectious Disease, Faculty of Medicine, 4615 Imperial College London, London SW7 2AZ, U.K.; ‡ Centre for Antimicrobial Optimisation, Imperial College London, Room 7S5, Commonwealth Building, Hammersmith Hospital Campus, Du Cane Road, London W12 0NN, U.K.; § Department of Electrical and Electronic Engineering, Faculty of Engineering, Imperial College London, Exhibition Road, London SW7 2AZ, U.K.; ∥ Department of Infection and Immunity, 8946 Imperial College Healthcare NHS Trust, St Mary’s Hospital, Praed Street, London W2 1NY, U.K.; ⊥ MRC Unit the Gambia at the London School of Hygiene and Tropical Medicine, Atlantic Boulevard, PO Box 273, Fajara, Banjul 00220, The Gambia; # 307955 Unité de Recherche Clinique de Nanoro, Institut de Recherche En Sciences de la Santé, Bp 218 Ouaga 11, Nanoro, Boulkiemdé 10010, Burkina Faso; ∇ The Fleming Initiative, Imperial College London and Imperial College Healthcare NHS Trust, St Mary’s Hospital, Praed Street, London W2 1NY, U.K.; ○ Department of Computing, Faculty of Engineering, Imperial College London, London SW7 2AZ, U.K.

## Abstract

Point-of-Care (POC) portable devices have shown significant potential for rapid pathogen identification in limited-resource settings. Colorimetric LAMP (cLAMP)-based POC nucleic acid testing is particularly promising due to its speed, sensitivity, and simple visual readout. However, there is a need for a universal, automated color detection method to improve ease of use and reduce readout subjectivity using portable devices. We proposed an AI-driven workflow for automatic detection of multiple pathogens using cLAMP in a multitube strip format. Following sample extraction, reagent rehydration, and reaction incubation, the cLAMP panel was photographed with a portable device. The algorithm performed: (1) image preprocessing to locate tubes; (2) segmentation and cleaning to extract the liquid-filled region using deep learning and Otsu's binarization, followed by pixel cleaning; (3) empty tube and invalid control checks; and (4) color detection using relative Maximum Mean Discrepancy (MMD; a nonparametric statistical distance between the hue distributions of two tube images) to identify samples. The AI workflow was evaluated on 1,636 photos from 527 tests for the differential diagnosis of three panels, for skin-tropic viruses, respiratory viral pathogens, and malaria parasites, under various lighting conditions in POC environments. The AI workflow was compared to expert-adjudicated human visual readouts as the reference standard. Rejection of invalid photos, defined as panels containing empty tubes or failed controls, achieved 98.94%, 98.29%, and 96.68% accuracy (95% CI 97.83−99.57%, 96.97−99.15%, 94.13−98.33%) for the three panels, respectively. Misclassified images were caused by human mistakes or extreme lighting conditions. Color identification on accepted images achieved 99.97%, 100%, and 99.69% accuracy (95% CI 99.82−100% for Data set 1 and 98.27−99.99% for Data set 3; one-sided 95% lower bound 99.90% for Data set 2). This study demonstrates a reliable automatic color readout workflow for cLAMP-based nucleic acid detection, showing potential for integration into POC testing apps for cloud-based use.

## Graphical abstract

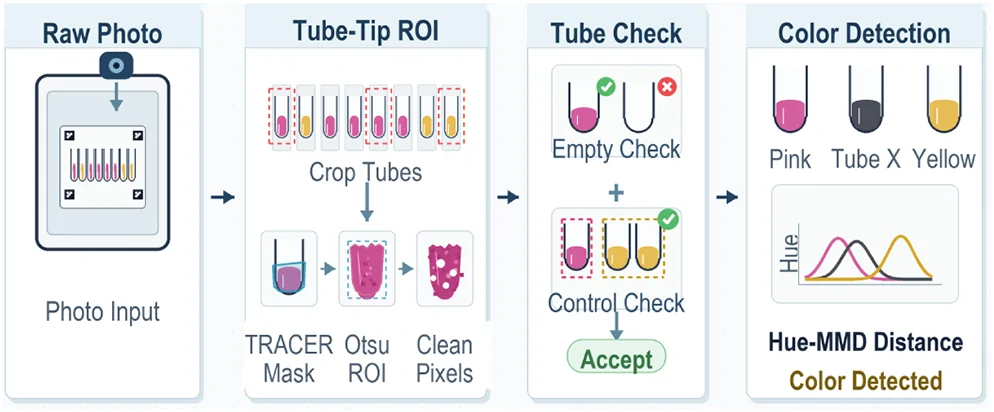

## 1 Introduction

Diagnostic testing plays a vital role in healthcare, yet a recent report from the Lancet Commission highlights that 47% of the global population still lacks access to essential testing.^1^ This underscores the urgent need for more affordable diagnostic solutions. Lateral flow tests (LFTs) have gained widespread use due to their simplicity, low cost, accessibility, and rapid results, though they generally offer lower sensitivity compared to molecular methods like polymerase chain reaction (PCR), the current gold standard.^2^ However, recent advances in portable molecular diagnostics have demonstrated great potential to deliver laboratory-grade sensitivity and specificity at the Point of Care (POC), whether in GP clinics, homes, or resource-limited settings such as rural health facilities.^3^

Among existing detection techniques for POC nucleic acid amplification tests (NAATs), loop-mediated isothermal amplification (LAMP) has garnered significant interest because of its fast time-to-result, high sensitivity, and isothermal incubation requirements, allowing testing to move away from bulky and expensive thermocycling instrumentation.^4^ However, LAMP-based tests often rely on complex optical equipment for fluorescent-based readouts, limiting their portability. Colorimetric LAMP (cLAMP) simplifies this by using visual color changes, allowing the detection of nucleic acid amplification based on pH-sensitive or metal ion indicators.^5^ This method has been proven effective for detecting pathogens such as SARS-CoV-2,^6–10^ influenza A, and other respiratory viruses,^11^ *Plasmodium falciparum*,^12^ *Aspergillus fumigatus*,^13^ *Campylobacter coli*,^14^ *Streptococcus agalactiae*,^15^ *S. mansoni* and *S. haematobium*,^16^ HCV,^17^ HIV and HPV,^18^ unlocking an easy-to-use, affordable, and simplified alternative technology to pathogen screening.

Despite its advantages, the reliance on naked-eye readouts for cLAMP presents new challenges. Human interpretation introduces subjectivity and errors, particularly under poor lighting conditions, in instances of color blindness or deficiency (with a reported prevalence of approximately 8% in men and 0.4% in women, varying across populations^19^), or when color differences are subtle or ambiguous,^20,21^ leading to low efficacy and precision of epidemiologic investigations.^8,9,14^ While expert visual interpretation under controlled conditions can be highly reliable, real-world POC testing is often performed by nonexpert users under variable lighting and operational constraints. Manual interpretation also imposes additional operational burden, requiring user training, attention, and manual labeling, which can slow decision-making and limit throughput in large-scale screening scenarios. The motivation for automated readout in this work is therefore not to replace expert judgment, but to deliver expert-level interpretation in a consistent, rapid, and user-independent manner. Such automation simplifies workflow, accelerates result reporting, and enables scalable, cloud-based aggregation of results for population-level surveillance and epidemiological analysis. Using standard camera technology on tablets or smartphones offers a low-cost solution to improve sensitivity and specificity in spotting positive samples, especially in situations of low initial target concentration and insufficient incubation. It also helps standardize automatic readouts to ensure reproducibility and facilitate multisite collaborations. In addition, certain camera-based technologies enable automatic color detection and target identification workflows, allowing for cloud-based automatic result reporting and integration into digital healthcare systems.^22,23^

However, automating cLAMP readouts presents its own challenges, mainly due to complex environmental factors such as ambient light conditions and dependency on device hardware.^9^ Current efforts focus on three approaches: **(1)** Custom-designed hardware platforms with built-in cameras/color sensors, usually combining the heating unit for incubation with the color readout units, which can either be built-in cameras^7^ or dedicated color spectrum sensors.^11,15,16^ The customized aspect of the approach (fixed sensor specifications and controlled lighting conditions in the enclosed reaction chamber) largely mitigates challenges in accurate color interpretation. However, large hardware costs detract from the use of the approach at the POC, particularly in limited-resource settings. **(2)** Portable devices (e.g., smartphones or tablets) leveraged in controlled environments^6,9,13,14,17,24,25^ with either an observation point for the direct observation with the camera chamber^14,24,25^ or an external component to repeatably align the camera with the sample tubes.^6,9^ In certain conditions where lighting and tube location are controlled, color detection can be tackled with conventional threshold-based image processing algorithms^13,14,25^ or ratiometric indexes^12,17^ which only work for limited platforms. **(3)** Portable devices used in noncontrolled environments that rely on advanced processing algorithms instead of specific hardware setups.^8,26^ Challenges like variable tube positions, shadows, overexposures, and inconsistent ambient lighting can greatly affect image segmentations and perceived color values in the RGB space^25^ invalidating traditional color thresholding and identification methods. Deep learning has been introduced to address these challenges, but existing works often require extensive training data sets that only work for the specific application scenario^8,26^ and raise concerns about interpretability and regulatory approval.^27,28^

To address key limitations of existing colorimetric LAMP readout approaches, namely reliance on subjective human interpretation, sensitivity to variable imaging conditions, and dependence on additional hardware or data-driven model training, this paper proposes a fully automatic cLAMP readout workflow with hybrid tube segmentation and color detection algorithms. It is validated with Dragonfly, a rapid, portable, and easy-to-use POC cLAMP-based molecular diagnostic platform, developed by our group^36^ and commercialized by ProtonDx Ltd.^30^ Dragonfly delivers accurate POC molecular diagnostics to resource-limited settings, simultaneously detecting and differentiating up to six distinct pathogen targets. However, the current platform relies on human interpretation for colorimetric readouts, offering opportunities to improve consistency and repeatability, as described above. In this context, automated colorimetric readout does not directly extend physical access to tests, but standardizes interpretation so that consistent, expert-level results can be obtained by nonexpert users wherever the portable device itself is available; the access gap is addressed upstream by the assay and the Dragonfly platform, while our workflow addresses the equally critical readout-consistency gap.

Dragonfly is paired with a companion app on a tablet, which captures an image of the end-point reaction tubes arranged in a custom card. The proposed AI-driven workflow then performs fully automated readout through a sequence of image processing, quality control, and color analysis steps, which are described in detail in the Results section.

Compared with existing approaches, the proposed workflow builds upon Approach (3) described above and is specifically designed to overcome several practical limitations:

- It avoids reliance on additional bulky hardware or controlled imaging setups, preserving portability and ease of use.
- It does not require application-specific training data for segmentation or color detection, reducing generalization risks across devices and environments. The deep-learning component (TRACER) is a generic, publicly released salient-object segmentation model pretrained on COCO; we use it as an inference-only building block, do not fine-tune it on cLAMP data, and combine it with deterministic image-processing and statistical checks that are fully auditable. No cLAMP-specific data set is used to optimize network weights, which differentiates our approach from prior deep-learning colorimetric readout pipelines trained on application-specific images.
- Its modular, rule-based decision logic enables transparent quality control and risk-aware rejection, aligning with medical device regulatory requirements. From a regulatory perspective, the pretrained segmentation network is treated as a locked, inference-only module whose output is subsequently filtered by deterministic morphological and statistical checks; this layered architecture isolates the data-driven component to a narrow, visually verifiable subtask (salient-region extraction) and preserves deterministic, traceable behavior for the clinically relevant color decision. The interpretability and regulatory implications of this design are discussed further in the Discussion.

Although image acquisition can be standardized using dedicated fixtures, fixed lighting, or enclosures, such approaches inevitably introduce additional hardware, setup steps, and user burden. In POC settings characterized by limited infrastructure, high throughput, and time pressure, these requirements can reduce compliance and scalability. The proposed workflow therefore intentionally avoids reliance on standardized image acquisition and instead focuses on algorithmic robustness to real-world variability.

To evaluate the performance of the proposed workflow under different conditions, three Dragonfly panels for different pathogens were used, generating three independent data sets. Data set 1 includes 67 tests with the Dragonfly Skin Infection Viral Test Panel (subset of^36^), and Data set 2 includes 129 tests using the Dragonfly Respiratory Test Panel (the panel presented in^30^). A total of 660 and 645 pictures were taken, respectively, for the two data sets using various portable devices, under different ambient lighting conditions. In addition, Data set 3 is included, with 331 pictures taken during malaria screening at the community level across four rural villages in Burkina Faso, using multiple devices and involving multiple users, in uncontrolled lighting conditions. Highly reliable performance was achieved, with invalid photo rejection accuracy of 98.94%, 98.29%, and 96.68%, respectively, for the three panels, and 99.97%, 100%, and 99.69% accuracy of color identification. This research demonstrated a reliable automatic color readout flow with broad applicability to cLAMP-based pathogen detection for infectious diseases, which could also be generalized for other colorimetric amplification chemistries. The approach carries a high potential for implementation in POC testing apps for cloud-based diagnostic flows.

## 2 Experimental Section

### 2.1 The Dragonfly Platform

Dragonfly Testing Kit (ProtonDx Ltd., United Kingdom) is a portable, sample-to-result molecular diagnostic platform designed for rapid multipathogen detection. Combining a simple power-free nucleic acid extraction method based on magnetic beads^31^ and lyophilized cLAMP technology,^32,33^ Dragonfly enables a rapid diagnostic solution without needing thermocycling, fluorescent readout, or cold-chain storage.

The algorithms described in this article are intended for integration into the Dragonfly Kit, providing an automatic solution from photo taking to result reporting. The details of the Dragonfly Kit are depicted in Figure S1, which includes a sample preparation kit, a test panel, a heating block, and the user app. Although developed on Dragonfly, the proposed algorithm workflow could be adapted for other diagnostic platforms and/or other amplification chemistries.

As depicted in Figure 1, after sample extraction, DNA/RNA is isolated, purified, and concentrated through the SmartLid method.^30,34^ SmartLid is a single-use cap that integrates magnetic-bead-based capture, wash, and elution steps into a manual rotation/actuation protocol so that extraction occurs in a closed tube without liquid handling. The two critical steps for downstream colorimetric readout are (i) sufficient elution volume to fully resuspend the lyophilized reagent pellets and (ii) complete removal of the bead-binding buffer before elution; both are enforced by the SmartLid geometry and are verified by the internal control tubes. The eluted nucleic acids are loaded into a panel of eight tubes, resuspending lyophilized cLAMP reagents. The first tube acts as a color control and remains pink (negative). The last two reactions function as the extraction and internal controls, respectively, and must be yellow (positive) for a valid test. Tubes 2–6 are the testing tubes and contain cLAMP assays that target five distinct pathogens. The panel is then incubated, unloaded, and placed into a Result Capture Card with a panel holder and four AprilTags in the corners.^35^ Chemical details of Dragonfly are further explained elsewhere.^36^

**Figure 1:**
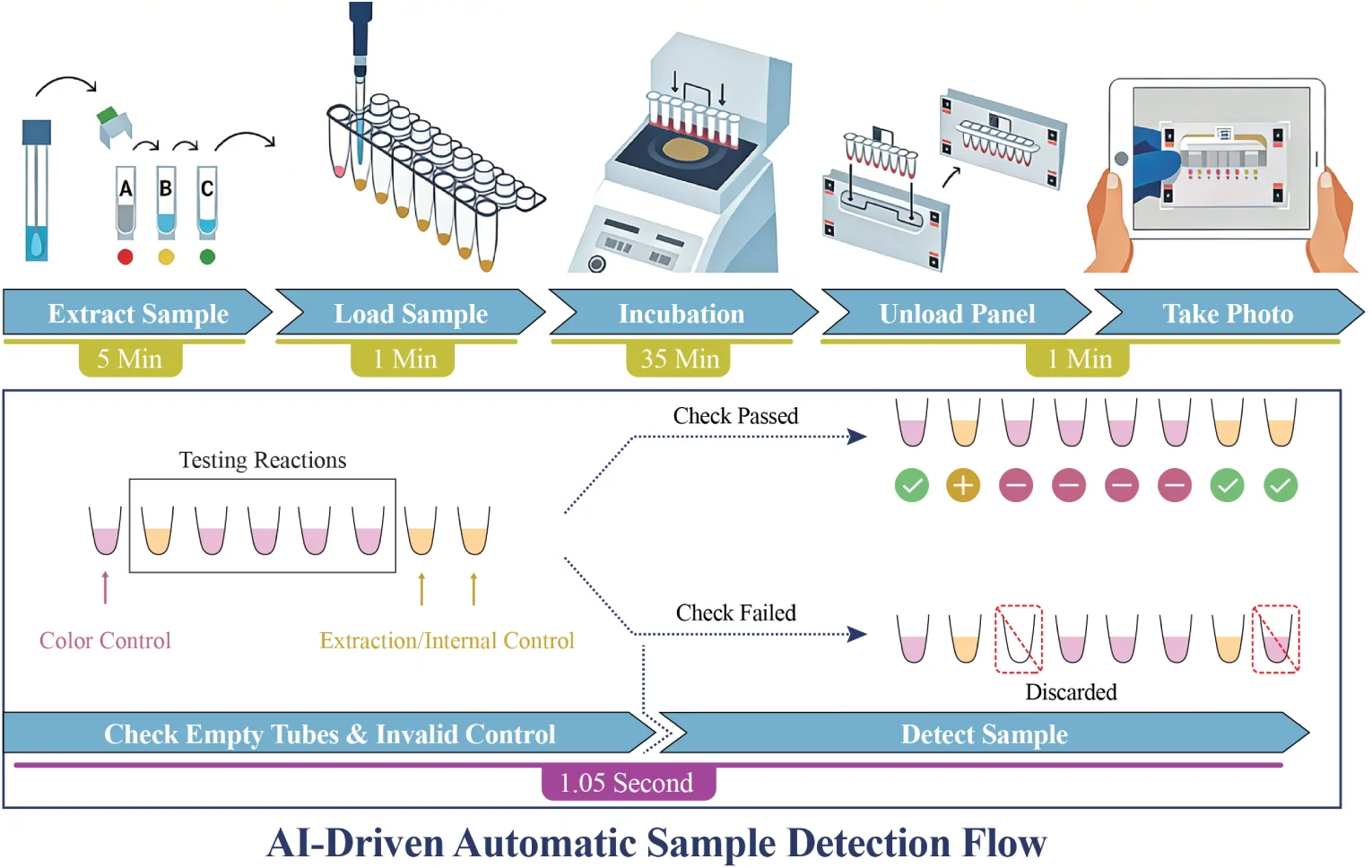
Dragonfly workflow with the proposed AI-driven readout flow. Samples are **(1)** extracted with magnetic bead-based extraction kits and **(2)** loaded into an 8-tube panel. The first and the last two tubes function as color and extraction/internal controls, and Tubes 2–6 target pathogens. The panel is then **(3)** inserted inside a dry bath heat block and incubated for 25 min at 63.5 °C. After the incubation, the panel is unloaded and **(4)** placed inside a Result Capture Card. **(5)** A tablet/smartphone app takes a photo of the card, to which the proposed AI-driven detection algorithms are applied. **(6)** Empty tube and control checks are then performed to exclude invalid panels. **(7)** Color identification runs on check-passed panels, outputting the detected color/testing result of each testing reaction tube. The AI workflow operates on Steps 5–7: the photograph captured in Step 5 is the input to Steps 6 and 7, which are the algorithmic contributions of this paper; all earlier steps are unchanged from the nonautomated Dragonfly procedure. At Step 5, the companion app displays a live camera preview with a Capture-Card alignment overlay; after capture, the app displays the captured image with tube positions highlighted for user confirmation before invoking the algorithm. At Step 7, the app presents the per-tube color classification as a panel-view with each tube color-coded (pink = negative, yellow = positive, gray = *Unknown*), the overall test result, and, where applicable, retake/repeat instructions when any tube returns *Unknown* or when the empty/invalid-control checks fail.

An integrated app, running on the portable device, guides the users through the testing process and captures a photo of the incubated panel placed on the Result Capture Card. Representative images and step-by-step illustrations of the Dragonfly platform and its companion app are provided in Figure S2, adapted from our previous system-level studies.^36,37^

After tube colors are identified, the final testing results are uploaded securely to the Cloud platform. There is no specific requirement for the device running the app, as long as a camera with >5 MP resolution is provided.

### 2.2 Data Sets Used in This Study

For validation purposes, we demonstrated the proposed algorithm flow on three test panels: Respiratory viral pathogens (for SARS-CoV-2, Influenza A virus, Influenza B virus, Respiratory syncytial virus, and Human rhinovirus), Skin-tropic viruses (for Orthopoxvirus, Monkeypox virus, Varicella zoster virus, Human herpesvirus 1, Human herpesvirus 2), and the Malaria panel. Examples of tests from the three panels are depicted in Figure S3.

Each test was categorized as positive (at least one positive reaction), negative (all testing reactions negative), empty (at least one tube is empty due to improperly loaded/placed strips), or invalid control (at least one control color is incorrect due to operation errors such as sample not loaded or incorrect sample extraction); representative examples of each category, drawn from all three panels, are shown in Figure S3 and referred to individually throughout the Results section.

*Sample-size structure*. Each test is one physical 8-tube panel run from an independent sample extraction; the 5–10 photographs per panel are technical replicates of the same completed reaction captured under different distances and lighting conditions. Accuracy is reported at the tube level with Clopper–Pearson confidence intervals. Figure 3d shows the photo-level rejection matrices, Figure 3e the tube-level color-classification matrices.

*Expert adjudication and inter-rater reliability.* Ground-truth labels for Data sets 1 and 2 were assigned by two trained molecular-diagnostics researchers (each with >5 years of experience reading cLAMP end-point readouts and blinded to algorithm output). Disagreements and panels tagged as ambiguous by either reader were resolved by a senior adjudicator (PhD molecular biologist, >10 years cLAMP experience) through side-by-side inspection of the full panel set for that test. The same two-reviewer-plus-senior protocol was applied to Data set 3. Inter-rater disagreement was concentrated in a small number of borderline ambiguous cases discussed in Discussion.

For the first two panels, we randomly selected two data sets from the available panel pool: 67 tests using the Skin Infection Viral Test Panel^36^ (33 positive, 28 negative, 1 empty, 5 invalid control) and 129 tests with the Respiratory Test Panel^30^ using spiked samples (52 positive, 58 negative, 7 empty, 12 invalid control). Each test panel was photographed 5 to 10 times using the built-in cameras of an iPhone 12 (12 MP) and an iPad Gen5 (8 MP) (Apple Inc., United States). To evaluate the robustness of the algorithm flow under various lighting conditions, photos were taken in a standard PCR lab with white ceiling lights, with each panel held vertically at approximately 20 cm, 40 cm, 60 cm, and 100 cm from the source, and 1–2 photos taken at each position. This results in 660 and 645 pictures for Data sets 1 and 2, respectively. An example picture captured is shown in Figure 2 “Raw Photo”. All photos were labeled by expert visual inspection (two trained adjudicators blinded to algorithm output, with senior adjudicator tie-breaking) as the ground truth for evaluation (not used for training). These labels were established through repeated expert inspection of each panel under multiple viewing conditions, not single-shot rapid judgments, in order to minimize the impact of variable lighting, operator variability, and visual perception limitations.

**Figure 2:**
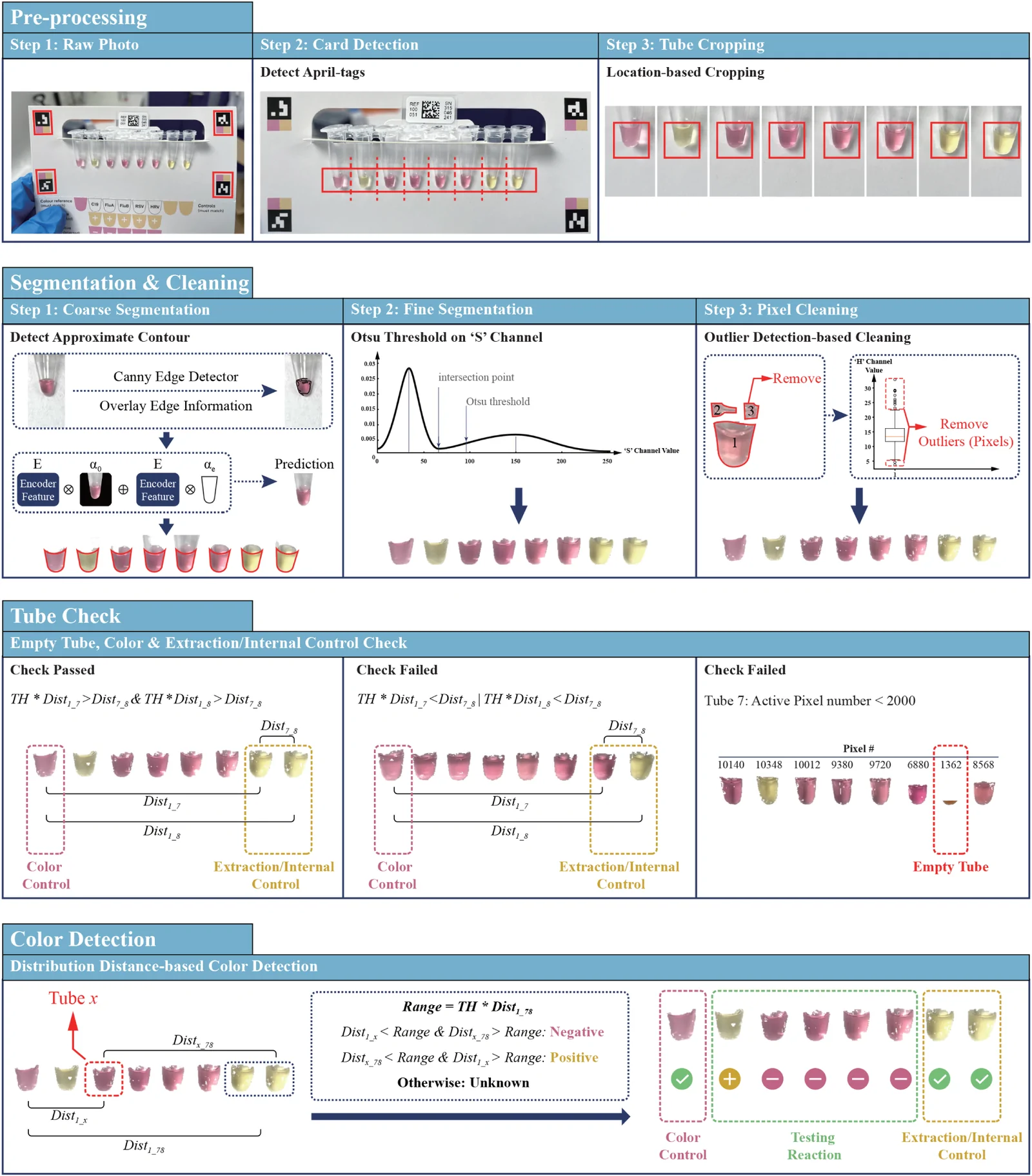
Proposed algorithm workflow. **Preprocessing:** Step 1 inputs a raw photo; Step 2 detects and adjusts the card location based on the AprilTags in the four corners; Step 3 crops the approximate images of tubes based on relative locations. **Segmentation & Cleaning:** Step 1 enhances the image with the Canny edge detector, and coarsely segments the tube area using a pretrained TRACER model; Step 2 finely segments out the solution-filled area by applying the Otsu’s binarization on the “*S*” channel of coarse segmented tube images; Step 3 recursively cleans the fine segmented images utilizing Connected Component Analysis, K-means clustering, and the Random Forest-based outlier removal algorithm. **Tube Check:** Check the existence of empty tubes with active pixel numbers and the correctness of control colors with MMD-based relative color distance comparison. **Color Detection:** Panels that pass the previous check are adopted with color detection by comparing the to-be-detected color with both negative and positive control colors and choosing the more similar one as output, under the scheme of relative MMD distance measuring of “*H*” values. The three segmentation steps (TRACER coarse segmentation → Otsu fine segmentation → recursive pixel cleaning) are applied strictly sequentially: there are no feedback loops between them; each step consumes only the output of the previous one. The two tube-check paths (empty-tube test via active-pixel count, and invalid-control test via MMD on hue distributions) are evaluated in parallel on each photograph; a photograph is accepted only if both pass, and any single failure triggers rejection. Figure 2 indicates the AND-logic between the two paths at the rejection node.

For the Malaria panel, 331 blood samples from community participants were tested and photographed on-site (outside health facilities, i.e., directly at community level), across four different rural villages in Burkina Faso by different operators from 25 November 2024 to 12 December 2024, using the built-in camera of Samsung Galaxy Tab S5e (13 MP, Samsung Electronics Co., Ltd.). No standardized guidelines were provided to the operators for capturing the photos, aside from the use of the Result Capture Card. This data set reflects the real-world situations of POC applications in the field, where challenging lighting conditions and operational variance exist. Since only one target pathogen is tested in this panel and reference tubes are reallocated, the following algorithm flow was adjusted accordingly when applied to this data set.

*Data set 3 details*. The 331 blood samples in Data set 3 were drawn from 331 unique community participants across four rural villages in the Central West Region of Burkina Faso. Participant-level infection status, cohort prevalence, and assay-level performance against a molecular reference are outside the scope of the present image-readout study and are not rederived here.

*Photography protocol in the field.* The absence of rigid photography guidelines was a deliberate design choice, intended to stress-test the algorithm under real deployment variability. Operators were instructed only to place the panel into the Result Capture Card and capture a full-frame image with the built-in camera; no distance, angle, or lighting constraints were imposed. Minimal user guidance (for example, an in-app alignment overlay) is flagged as a deployment improvement in Discussion.

*Spiked-sample preparation (Data set 2).* Respiratory-panel spiked samples were prepared by spiking commercially available inactivated viral particles (Vircell or AB Scientific) for each target pathogen into inactivating Molecular Transport Medium (eNAT, Copan) at concentrations spanning the clinically relevant range for each target (typically 10^3^–10^5^ copies/mL, calibrated per stock per batch). Because the end-point is a bulk pH-indicator change, matrix effects between spiked transport medium and clinical respiratory samples are expected to be small.

The spiked samples were prepared by spiking commercially available viral particles into inactivating Molecular Transport Medium (MTM) to enable controlled performance evaluation, whereas anonymized samples were derived from real clinical patient specimens collected under ethical approval.

*Reconciling the human reference with the stated motivation.* We validate the algorithm against the best currently available human reference: two trained adjudicators with senior tie-breaker, working from the same images under controlled conditions. This is complementary to, not in conflict with, the goal of reducing routine user subjectivity at the point of care. The question of whether the algorithm can detect subtler color shifts than human readers is treated separately in Discussion and the supplementary early incubation experiments (Figure S4).

## 3 Algorithm Flow for Automatic Color Detection and Target Identification

The algorithm flow processes the raw photos captured by the Dragonfly App, delivering pathogen detection results or rejecting photos based on empty tubes or invalid controls. Figure 2 depicts the algorithm flow we proposed, which contains four parts: image preprocessing, tube segmentation and pixel cleaning, tube check, and color detection. All the algorithms were developed and evaluated on a workstation (CPU Intel Xeon Gold 6226R * 2, RAM 128G, GPU NVIDIA RTX A5000) with Python 3.9.17. Code implementing the proposed workflow, together with a demonstration data set, is publicly available at^38^ to support reproducibility.

### 3.1 Image Preprocessing

The user holds the Result Capture Card in front of the camera which captures the raw image if at least three AprilTags are detected. AprilTags are special black-and-white markers originally designed for robotic applications, enabling fast target location identification.^35^ Three tags, not all four, are required because three noncollinear points are the geometric minimum needed to recover the planar homography of the card; requiring four would fail if a single tag is obscured by a finger or glare, while requiring only two is mathematically underdetermined and would admit spurious rectifications. If fewer than three tags are detected, the photograph is rejected before any downstream processing and the user is asked to recapture. Step 2 involves detecting the four corners of the card based on the identified AprilTag locations, applying trapezoidal rectification (which corrects card-plane perspective distortions within the range of out-of-plane tilts encountered during normal hand-held use; beyond a moderate tilt angle, the AprilTag detector typically fails first, at which point the photograph is rejected upstream by the three-tag rule above and is not subjected to a potentially spurious rectification), and resizing the image. After this step, the card is cropped into a fixed size of 2000 × 500 pixels.

The Result Capture Card is designed to hold the tubes at a constant *X*-axis position, while there may be *Y*-axis variations depending on user operations. Therefore, Step 3 crops the approximate location of the tubes by dividing the designated *X*-axis locations of the card while keeping a relatively large *Y*-axis range. In most cases after this step, each cropped image will contain one tube and surrounding background pixels, making it ready for tube segmentation in the next step.

### 3.2 Tube Segmentation and Pixel Cleaning

Although an approximate location of the tube is known from the previous step, it is not practical to use all the pixels of the tube image and classify color values, since the background pixels may contain shadows and light spots which can affect color value distributions.^25^ Furthermore, certain pixels in the solution-filled area may be influenced by reflections under extreme lighting situations. Therefore, the fine contour of the solution-filled area of the tube (tube tip) needs to be depicted and the tube tip needs to be segmented and cleaned. To achieve this goal, three steps are involved.

First, the tube must be segmented from the background (coarse segmentation). Since the textural features of the background are complex and conventional color-based thresholding may not work properly, we adopted a state-of-the-art deep learning model, TRACER_B7, which has been pretrained on large natural image data sets^39,40^ to identify the tube as an object. TRACER_B7 was chosen because it is a publicly released salient-object segmentation network trained on large generic image data sets and, crucially for our use case, produces clean binary masks of high-contrast objects without requiring fine-tuning; we compared it informally against U-Net and Mask R-CNN alternatives during early development, but those models are either designed for application-specific training (and therefore reintroduce the training-data requirement we explicitly want to avoid) or operate at resolutions that do not fit the 2000 × 500 strip layout without padding artifacts. Before inputting the tube image into the network, a Canny Detector with threshold 1 as 15 and threshold 2 as 30 was applied and added to the picture to emphasize the edge information and mitigate the impact of out-of-focus blur on object contour, generating the enhanced pictures.^41^ The two Canny thresholds (15 and 30) were chosen to follow the widely recommended 1:2 ratio and are conservative defaults that pass strong edges while suppressing noise; they were not optimized on the three evaluation data sets. This distinction between a fixed hyperparameter choice and data-driven parameter learning is important: no gradient-based optimization is performed against any labeled subset of these data, which is the meaningful sense in which the workflow is training-free. The TRACER detects objects using attention-guided tracing modules incorporated with explicit edges.^39^ With enhanced inputs, this pretrained model enables precise tube identification without the need for additional data labeling, thereby facilitating the transition to new scenarios and avoiding issues with generalization.

Second, the TRACER model can roughly segment the tube from the background, yet the output of the network may contain both the empty and the solution-filled parts of the tube. Also, the segmentation details can be affected by ambient factors such as reflections. Therefore, fine segmentation is required as the second step to further narrow the ROI into merely the tube tips where pixel colors are pure. The HSV color space represents colors using three components: Hue (*H*), which encodes the dominant wavelength as an angular value; Saturation (*S*), which describes color purity or intensity; and Value (*V*), which corresponds to overall brightness. For color-based operations, it has been proven that using HSV space, which linearly represents the hue with only one value, can effectively eliminate the lighting-induced color value variations when using commercial cameras in ambient environments.^7^ Therefore, we first transformed the coarse-segmented image from RGB to HSV space.^42^ We then used the “*S*” channel value to represent the image as a grayscale picture, depicting the color saturation. Given the assumption that the empty area of the tube is more transparent and less saturated compared with the filled tip, Otsu’s binarization was used to fine-segment the tube tips by finding a threshold *k* in the “*S*” channel histogram which minimizes the following weighted within-class variance:^43^

$$\sigma^{2}=p_{A}\left(k\right)p_{B}\left(k\right){\left(m_{A}\left(k\right)-m_{B}\left(k\right)\right)}^{2}$$

where

$$\begin{array}{cc} p_{A}\left(k\right)=\overset{k}{\underset{i=0}{\sum }}p_{i}, & p_{B}\left(k\right)=\overset{255}{\underset{i=k+1}{\sum }}p_{i},\\ m_{A}\left(k\right)=\frac{\overset{k}{\underset{i=0}{\sum }}ip_{i}}{p_{A}\left(k\right)}, & m_{B}\left(k\right)=\frac{\overset{255}{\underset{i=k+1}{\sum }}ip_{i}}{p_{B}\left(k\right)} \end{array}$$

*p*_*i*_ is the probability of a pixel of grayscale value *i*, *p*_A_(*k*) and *p*_B_(*k*) are the probabilities of a pixel to be categorized into Group A (pixel value < *k*) and Group B (pixel value > *k*), and *m*_A_(*k*) and *m*_B_(*k*) are the mean values of the two groups. The optimal *k* was found by applying a grid search in [0,255].

Finally, after applying the fine segmentation, pixel cleaning was adopted to remove the abnormal pixels passed from the previous steps which may be caused by light spots or reflections on the tube wall. The detailed algorithm for pixel cleaning is illustrated in Table [Table utbl1] and Figure 2 Segmentation & Cleaning Step 3. The RGBA image was first transformed into HSVA space, following which a connected component analysis^44^ was introduced to collect the connected areas of active pixels (where transparency channel “*A*” value = 255) and keep only the two largest connected components (e.g., in the figure, the third-largest component is removed). After that, recursive steps of K-means two-clustering on “*S*” channel values and removing the cluster with the smaller “*S*” channel median value were applied, until the total number of active pixels was smaller than 14,000, corresponding to the expected area of a tube tip region in the normalized images. This threshold is therefore determined by the physical geometry of the tube and card design, not by the native camera resolution. If tube geometry or placement were modified, this parameter could be adjusted accordingly. Finally, an Isolation-Forest-based outlier removal algorithm^45^ with a 20% contamination ratio was adopted on “*H*” values of active pixels to remove the outlier pixels that have significant differences with the majority. After pixel cleaning, the remaining active pixels should be suitable for color detection in the following steps. The 20% contamination ratio is a conservative value in line with commonly adopted practice in image-based outlier-detection literature (typical range 10–25%), chosen to remove reflective artifacts, boundary-adjacent pixels and localized shadows while preserving the bulk of genuine reaction pixels. Sensitivity checks within this typical range indicate that the algorithm’s headline accuracies are not dominated by the exact choice of contamination ratio. The pixel-cleaning sequence (K-means on *S* to remove desaturated background-like pixels, then Isolation Forest on *H* to remove hue outliers) is designed to preserve genuine within-tube color variation. Because the subsequent color decision is made on the distribution of retained *H* values, not on their mean, a partially mixed reaction with a broad but internally consistent hue distribution is not mistakenly discarded; empirically, the mean *H* value of the retained pixels is very close to the mean H of all TRACER-accepted pixels, indicating that cleaning narrows the distribution without shifting it.

**1 utbl1:** Pixel-Cleaning Workflow

1	Input image in *R*, *G*, *B*, *A* matrix
2	*R*, *G*, *B*, *A* → *H*, *S*, *V*, *A*
3	Descending ranked *N* components ← Connected Component Analysis on active pixels (*a* = 255 in *A*)
4	If *N* > 2: Remove pixels (*a* ← 0) ∈ Component 3, 4, ..., *N*
5	While number of *a* = 255 in *A* > 14,000:
6	Cluster index *c*_1_,*c*_2_ ← Apply K-means clustering on *S* values where pixel *a* = 255 in *A*
7	If median $\left(S_{c_{1}}\right)$ > median $\left(S_{c_{2}}\right)$ : remove pixels ∈ *c*_2_
8	Else: remove pixels ∈ *c*_1_
9	End While
10	Apply 20% Isolation Forest on *H* values where pixel *a* = 255 in *A*

### 3.3 Tube Check

Before identifying the solution colors, the algorithm must check whether empty tubes or invalid controls exist and decide whether the photo should be rejected. Empty tubes were easily picked out and discarded from further analysis if any tube showed an active pixel number smaller than 15% (approximately 2,000 in this research) of the normal pixel number for a tube after pixel cleaning. In our data sets, the per-tube active-pixel count for correctly filled tubes is comfortably above this cutoff, so a valid but slightly underfilled tube retains a safety margin before being flagged as empty; the cutoff is therefore well separated from the operating range of valid tubes.

We then define the color distance between Tube *x* and Tube *y* as:

$${\mathrm{Dist}}_{x\_y}={MMD(H_{x},H_{y})}^{2}$$

The squared form *MMD* ^2^ is used in place of *MMD* itself because the kernel-based estimate of MMD is naturally expressed as a sum of inner products and is always non-negative in its squared form, making it directly interpretable as a statistical distance and computationally cheaper (no square root). The squared value preserves the ordering used in all subsequent inequalities so no decision is affected by this choice.

$${MMD(H_{x},H_{y})}^{2}=\frac{1}{m^{2}}\overset{m}{\underset{i=1}{\sum }}\overset{m}{\underset{j=1}{\sum }}k\left(h_{x_{i}},h_{x_{j}}\right)+\frac{1}{n^{2}}\overset{n}{\underset{i=1}{\sum }}\overset{n}{\underset{j=1}{\sum }}k\left(h_{y_{i}},h_{y_{j}}\right)-\frac{2}{mn}\overset{m}{\underset{i=1}{\sum }}\overset{n}{\underset{j=1}{\sum }}k\left(h_{x_{i}},h_{y_{j}}\right)$$

$$k\left(h_{1},h_{2}\right)=\cos\left(\frac{\pi }{360}\cdot d\left(h_{1},h_{2}\right)\right)$$

$$d\left(h_{1},h_{2}\right)=\min\left(\left|h_{1}-h_{2}\right|,360-\left|h_{1}-h_{2}\right|\right)$$

where $H_{x}={[h_{x_{i}}]|}_{i=1,...,m}$ and $H_{y}={[h_{y_{j}}]|}_{j=1,...,n}$ are the active pixel column vectors of Tube *x* and *y* in *H* values. Following the concept of Maximum Mean Discrepancy (MMD)^46^ and considering the nature of HSV space as a cylindrical coordinate system, we defined a new kernel function *k*(*h*_1_,*h*_2_), called **Period-Linear Kernel**, which calculates the minimal angle on the circle of *H* values as the intercolor distance *d*(*h*_1_,*h*_2_) and maps it to the range [0,1].

Hue values in the HSV color space represent angular coordinates on a circular domain, not linear values in Euclidean space. Consequently, conventional kernels defined on linear spaces (e.g., Gaussian or Laplacian kernels) are not suitable for measuring hue similarity, as they fail to account for the periodic nature of the hue dimension.

The Period-Linear Kernel was therefore introduced to explicitly model the minimal angular distance between hue values on the circular domain, enabling meaningful comparison of hue distributions while preserving periodicity. The key issue is that hue is a circular variable, so formulations that do not respect periodicity are not appropriate for this task in the first place. The Period-Linear form was selected because it explicitly respects the circular geometry of hue while remaining simple to interpret (linear mapping of minimal angular distance to [0,1]) and lightweight to compute.

Therefore, Dist_*x*__ *_y_* is a measurement of the *H* value distribution discrepancy between the two tubes. Smaller Dist_*x*__ *_y_* indicates more similar colors in tubes.

The following criteria are used to check whether control tubes show the correct colors:

$$\mathrm{TH}\;*\;{\mathrm{Dist}}_{1\_7}>{\mathrm{Dist}}_{7\_8}\;and\;\mathrm{TH}\;*\;{\mathrm{Dist}}_{1\_8}>{\mathrm{Dist}}_{7\_8}\to Check\;Passed$$

$$\mathrm{TH}\;*\;{\mathrm{Dist}}_{1\_7}<{\mathrm{Dist}}_{7\_8}\;or\;\mathrm{TH}\;*\;{\mathrm{Dist}}_{1\_8}<{\mathrm{Dist}}_{7\_8}\to Check\;Failed$$

where *TH* is an adjustable coefficient for tuning the algorithm sensitivity and is fixed at 0.3 in this research. Although values in the interval 0.2–0.3, including 0.25, are feasible on the present data sets, we wanted to set a single value that could be cross-validated across multiple data sets. We therefore selected *TH* = 0.3. The theoretical interpretation of *TH* is a relative tolerance on the dispersion of the Tube 7–8 pair when compared against the Tube 1–7 and Tube 1–8 separations; a fraction of the control–control distance is a natural scale because the same imaging conditions affect both numerator and denominator and the ratio is, by construction, lighting-invariant. We therefore multiply Dist_1_7_ and Dist_1_8_ by *TH* instead of a direct comparison. As we mentioned before, Tube 1 serves as the color control and should always be pink, whereas Tube 7 and 8 are Extraction and Internal Control and should always be yellow. The criteria require the distance between Tube 7 and 8 (which should be very small) much smaller than both the Tube 1–7 and Tube 1–8 distances (which should be very large). If any criterion is not satisfied, the algorithm considers the controls incorrect and rejects the photo. Instead of using fixed color value ranges to define colors, the proposed criteria measure relative color differences and make judgments based on the chemical nature of the reactions, to avoid the potential color value shifts caused by lighting conditions or camera specification discrepancy.

### 3.4 Color Detection

Images that successfully passed the tube check would be accepted for the final color (target) detection. We followed the same concept of relative color distances in the detection step, setting the criteria below for Tube *x*:

$${\mathrm{Dist}}_{1\_x}<\mathrm{Range and}{\;\mathrm{Dist}}_{x\_78}>Range\to Negative$$

$${\mathrm{Dist}}_{x\_78}<Range\;and{\;\mathrm{Dist}}_{1\_x}>Range\to \mathrm{Positive}$$

Otherwise→Unknown

where

$$Range=\mathrm{TH}\;*\;{\mathrm{Dist}}_{1\_78}$$

Dist_1_78_ indicates the MMD distance between the *H* values of cleaned Tube 1 and the combined *H* values of cleaned Tube 7 and 8. To classify Tube *x*, the algorithm checks, respectively, its *H* values’ distance with the standard color Pink (Dist_1__ *_x_*) and Yellow (Dist_*x*_78_). If one of the two distances is smaller than Range and the other one is not, Tube *x* must have a very similar color to the corresponding standard color; thus, a decision can be made. Otherwise, Tube *x* is not close to either of the standard control colors, and an “Unknown” result will be exported. By changing the coefficient *TH*, the strictness of the decision can be adjusted, enabling a controllable decision-making process which is required for medical-grade AI algorithms. The linear scaling of the acceptance range with Dist_1_78_ follows directly from the relative-distance design: the pair (Tube 1, Tubes 7–8) provides an in-photo, in-panel reference for how far two known colors are in the current image, and scaling the acceptance radius with this distance makes the decision invariant to any multiplicative shift of the hue distribution (which is the dominant failure mode under variable lighting). A nonlinear scaling would require an external calibration curve and would reintroduce the data set-specific tuning we are trying to avoid. The behavior of the algorithm as *TH* is varied, showing the tradeoff between over- and under-rejection, is presented in Figure S6; the plateau *TH* ∈ [0.2, 0.3] corresponds to the operating region in which both rejection and color-classification accuracies remain near their peak on all three data sets, and the two plateau end-points are not statistically distinguishable on paired tube-level calls (McNemar’s test, nonsignificant at the 5% level; see Discussion). When the algorithm returns *Unknown*, the in-app guidance instructs the user to first retake the photograph under different lighting (which often resolves Unknown calls in our data sets) and, if the result remains *Unknown*, to repeat the test; this workflow is documented in the companion app and flagged in Discussion.

Once the proposed algorithm flow is integrated into the app, the detected results for the five pathogens can be presented to the user through the software, which will also give feedback and instructions on any of the aforementioned exceptions (empty tubes, invalid controls), allowing the user to redo the experiment or retake the photo from the initial step.

## 4 Results

The qualitative and quantitative performance evaluations of the proposed algorithm flow are provided in Figure 3. Note that for the Malaria panel, the color, extraction, and internal controls are located in Tube 1–3, respectively, and only one test is conducted in Tube 4, so the steps of Tube Check and Color Detection were adapted accordingly following the same concept.

**Figure 3:**
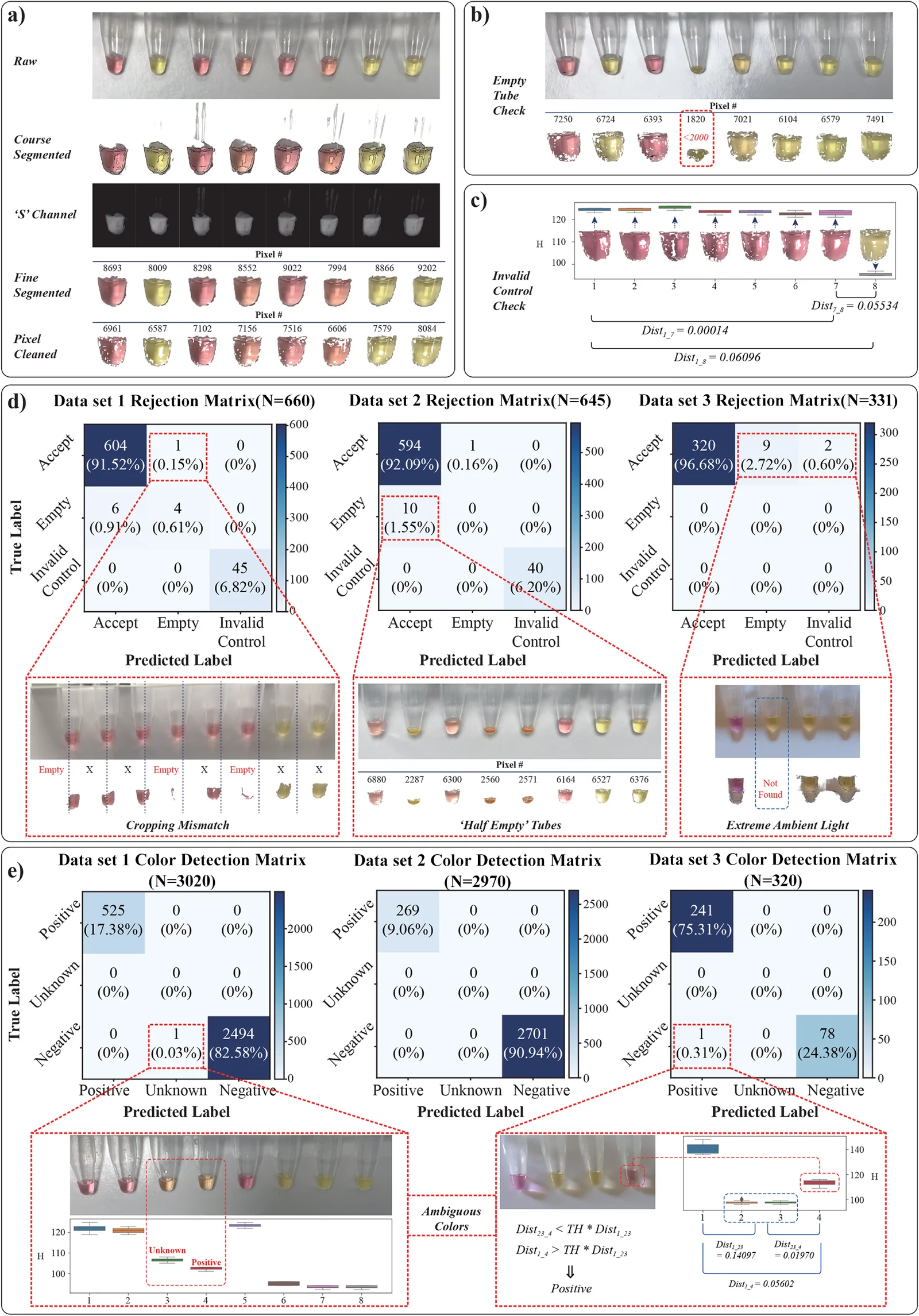
Results on the three data sets. **a)** A demonstration of tube segmentation and pixel cleaning, showing the raw image, the coarse segmented tubes and their corresponding “*S*” channel grayscale images, the fine segmented tubes, and tubes after pixel cleaning on each row, respectively. **b)** A panel with an empty tube (Tube 4), in which the active pixel number is smaller than the empirical threshold of 2,000. **c)** An example where incorrect extraction control occurs, with boxplots of “*H*” values shown. MMD distances Dist_1_7_, Dist_1_8_, and Dist_7_8_ are presented. **d)** The rejection matrix of Data sets 1–3 depicting the quantitative performance of the empty and control check. Numbers in the matrices indicate the number of panel photos of the corresponding category. Three examples of incorrectly rejected photos are illustrated, in which cropping mismatch, “Half-empty” issue, or extreme ambient lighting occurs. **e)** Confusion matrices of color/target detection performance on accepted panels (panels that belong to the upper-left corners of rejection matrices) for Data sets 1–3, with misclassified samples presented, in which ambiguous colors exist. Under typical conditions, imperfect coarse TRACER masks were often recovered by the downstream Otsu and connected-component stages; more difficult cases arise mainly under extreme or limiting conditions. The 2,000-pixel cutoff in panel b lies well below the operating range of correctly filled tubes, and panel c boxplots use the OpenCV HSV hue range [0, 180]. Panels **d** and **e** report observed rejection and color-classification performance across the three data sets.

### 4.1 Examples of Tube Segmentation and Pixel Cleaning

A demonstration of tube segmentation and pixel cleaning is illustrated in Figure 3a, where the raw image, the coarse-segmented tubes and their corresponding “*S*” channel grayscale images, the fine-segmented tubes, and tubes after pixel cleaning are presented. The background of the raw image contains complex textural features due to the poor ambient lighting, but the TRACER network with enhanced inputs can still roughly extract tube locations and contours, providing smooth edges. However, since the model extracts context-based information, it also includes some transparent parts of the tubes affected by complex ambient lighting. Analyzing the “*S*” channel of the HSV space reveals significant differences between empty and filled tube areas, where solution-filled areas have higher “*S*” values and look brighter. This creates a bimodal distribution for “*S*” values of active pixels and benefits the further step of Otsu’s binarization. The Otsu’s threshold successfully separates the background and ROI, outputting clean tube tips with almost no empty parts. After pixel cleaning, approximately 15–20% of outliers with extreme “*H*” values are removed, resulting in slightly fewer pixels and blank points in the ROI, as shown in Figure 3a Row 5. Although the tube tips may look “broken” after pixel cleaning, the algorithm narrows the “*H*” value distribution, purifying the color hue, and making the following distance measurement more accurate. The “broken” appearance is a thinning, not a shift, of the tube-interior pixel set: boundary-adjacent pixels (which have systematically different optical properties due to tube-wall refraction and glare) are the ones preferentially removed, so the color measurement becomes *less* edge-biased. Qualitatively, the in-tube standard deviation of *H* is substantially reduced after cleaning, while the mean *H* shifts only marginally, indicating that the procedure narrows the hue distribution without translating it; this is the intended behavior for the downstream MMD-based distance comparison. Across the three data sets, TRACER coarse segmentation could fail in a small number of cases, typically under extreme or limiting conditions; when the coarse mask was imperfect, the downstream Otsu and connected-component stages often recovered a usable tube-tip region for the subsequent color analysis.

### 4.2 Demonstrations of Empty Tubes and Invalid Controls

Figure 3b illustrates a panel with an empty tube, which can be caused either by operation errors or by evaporation promoted during long-term storage. As the active pixel number for Tube 4 is smaller than 15% of the normal pixel number for a tube (approximately 2,000), this photo is rejected, and the user will be asked to rerun the experiment.

Figure 3c demonstrates an example of invalid extraction control. By examining the “*H*” value boxplots of cleaned tube images, similar distributions with close median values can be seen among the same color, while different colors exhibit large discrepancies. MMD distances further verify the concept that color-value distribution differences function as a sensitive and reliable metric for differentiating colors, with Dist_1_7_ being extremely smaller than Dist_1_8_ and Dist_7_8_. We do not interpret the statistics of the absolute distance distributions as especially meaningful here, because the workflow is based on relative distance rather than absolute distance. Under different lighting conditions, these absolute distances can vary substantially, so comparing them directly does not provide a useful interpretation; the relevant quantity is the within-panel relative comparison used by the decision rule.

### 4.3 Quantitative Performance of Empty-Tube and Invalid-Control Check

Figure 3d shows the quantitative performance of the empty tube and invalid control check on the three data sets. The rejection matrices show how the algorithm reacts to exceptions. Numbers in the matrices indicate the number of panel photos of the corresponding category. Most photos are correctly classified and rejected if abnormal, with rejection accuracy of 98.94%, 98.29%, and 96.68%, respectively (Clopper−Pearson 95% CI 97.83−99.57%, 96.97−99.15%, 94.13−98.33%). Of the panels with ground-truth-valid controls, the empty-tube/invalid-control check produced 6, 10, and 0 false *acceptances* (0.91%, 1.55%, and 0% rate, respectively) and 1, 1, and 11 false *rejections*.

In Data sets 1–3, a few images which should be accepted are predicted as empty tubes or invalid controls existing. Certain false rejections will not affect the accuracy of color identification (because they are excluded from the following steps) but can slightly increase the false rejection. Two typical reasons for these false rejections are cropping mismatch and extreme ambient light, as illustrated in Figure 3d lower part. A qualitative post hoc review of the Data set 3 false rejections suggests that the most commonly observed failure modes were cropping mismatch and extreme ambient light. Across the three data sets, these same two categories recur most often among the interpretable false-rejection cases, with some cases attributable to a combination of the two or left unattributed from the image evidence alone. Cropping mismatch is directly targetable by in-app placement guidance, while extreme lighting is potentially reducible by guided recapture prompts. Cropping mismatch occurs when the panels are not correctly placed into the Capture Card (e.g., tilted as shown in the figure), resulting in tubes being cropped by half and fewer active pixels. This situation can be solved when the user gets corresponding App feedback and readjusts the panel placement. Extreme ambient light conditions, such as strong spotlight sources or insufficient lighting, can compromise tube detection. Figure 3d illustrates an example where shadows caused by insufficient lighting blur the boundaries between the tube and background. Such exceptions can be resolved by taking another photo with a different lighting condition as instructed by the app.

The rejection matrices of Data sets 1 and 2 show tubes considered empty by the ground truth are accepted by the algorithm. This is due to the “Half-empty” issue, as demonstrated in Figure 3d, where the reactions are completed but there is less solution. The algorithm’s decision rule operates on the active-pixel hue distribution, not on perceived fill level, so a tube with reduced but nonzero reagent volume is evaluated on its color content, not on its fill level; the human reviewer, in contrast, conservatively flags low-volume tubes as “too empty to trust”. In this narrow regime, the two judgments are not strictly contradictory but apply different operational thresholds, and the Discussion frames the algorithm’s behavior as a sensitivity feature, not an error. A formal reconciliation using an instrument-grade volume reference would require additional experiments and is proposed as future work. This issue did not affect color detection accuracy in our data set, as all incorrectly accepted tubes were still correctly classified by color. Further discussion is provided in the later discussion of misclassification consequences.

The acceptance rate across data sets is around 95.6%, indicating that high color-identification accuracy was achieved without excessive rejection. The resulting repeat rate of approximately 4.4% is operationally acceptable for clinical deployment, because the workflow trades a small number of repeats for a substantial reduction in the risk of incorrect human end-point calls on questionable panels. In a cloud-enabled deployment, many repeats can be triggered by the app from the captured image alone, without requiring a fresh panel.

*Statistical analysis note.* All accuracy figures reported in the following subsections are accompanied by Clopper–Pearson exact 95% confidence intervals computed at the tube level. Figure 3d reports the photo-level rejection matrices and Figure 3e the tube-level color-classification matrices. The within-test clustering is expected to be modest because the 5–10 technical replicates of the same completed reaction are largely independent draws of the imaging noise, not of the underlying reaction outcome.

*Between-data set comparison of rejection accuracy.* A χ^2^ test on the 2 × 3 table of correctly/incorrectly classified photographs across the three data sets (χ^2^ = 6.51, d*f* = 2, *p* = 0.039) provides evidence against the null hypothesis of a common rejection accuracy; the observed differences (98.94% / 98.29% / 96.68%) indicate between-data set heterogeneity.

### 4.4 Performance of Target Detection

Figure 3e provides the color classification accuracy of the three data sets in confusion matrices. The numbers in the matrices indicate the numbers of testing reaction tubes of accepted photos (photos categorized into the top-left corners of rejection matrices). The proposed algorithm achieved 99.97%, 100%, and 99.69% accuracy of color identification, respectively (Clopper−Pearson 95% CI 99.82−100% for Data set 1 and 98.27−99.99% for Data set 3; one-sided 95% lower bound 99.90% for Data set 2), with only two out of 6,310 tubes (3,020 in Data set 1, 2,970 in Data set 2, 320 in Data set 3) misclassified. The two wrongly detected samples are presented in Figure 3e lower part, both of which showed ambiguous colors. Boxplots also show “in-between” distributions of pixel colors. We do not claim the algorithm is strictly “wrong” on these ambiguous tubes: expert adjudicators placed them on the pink side of the canonical color space, while the algorithm’s relative-distance measurement placed them closer to the yellow side. Independent objective measurements (spectrophotometry or pH meter) were not available for these specific tubes, and resolving such borderline cases with an instrumental reference is proposed as future work. The single Data set 3 misclassification was a *false positive* relative to the expert visual adjudication reference, i.e., a pink tube classified as yellow by the algorithm. The interpretation is therefore limited here to agreement against the expert end-point-readout reference used in this study. A qualitative post hoc inspection of the Data set 3 photographs suggests that low luminance and low sharpness were the two most commonly observed photo-quality factors associated with rejection, consistent with the cropping-mismatch and extreme-lighting failure modes described above. For the “Half-empty” accepted-by-algorithm tubes, all tubes produced a color classification that agreed with the expert reading of the same photograph at normal viewing; we therefore frame this regime as algorithm sensitivity to a valid-but-low-volume tube, not as an algorithm error, as now discussed in Discussion. Further discussion is provided in the later discussion below.

## 5 Discussion

### 5.1 Sensitivity to Ambiguous Colors

As depicted in Figure 3e, in some situations, tubes may exhibit ambiguous colors between pink and yellow, which can be unfinished reactions caused by insufficient incubation time. To explore the algorithm response to this abnormal situation and evaluate the method’s sensitivity, we carried out supplementary experiments using Skin-tropic virus panels with three concentrations, taking photos every 5 min after incubation. The color-transforming phases were spotted, and the algorithm performed robustly with high sensitivity in abnormality detection when ambiguous colors showed up, as long as the *TH* is set inside a reasonable range. Technical details and results can be found in Figure S4, Figure S5, and Section S1 in the Supporting Information. Briefly, in those experiments, the algorithm at *TH* = 0.3 detected the onset of color change earlier than the canonical end-point used for clinical reading, with the same panels rated as “unclear” by adjudicators at the matching time points. This further highlights the value of automated readout workflows in sensitively capturing subtle color changes under conditions of low initial target concentration or insufficient incubation. Through adjustment of the decision threshold, such quantitative sensitivity may support earlier discrimination of evolving reactions, without requiring premature binary classification.

In such borderline cases, discrete ground-truth assignment itself becomes inherently uncertain, and occasional disagreement between expert-adjudicated labels and algorithmic output reflects this ambiguity and not a failure of the validation strategy.

Supplementary experiments with insufficient incubation time also indicate that the algorithm can, in some cases, detect subtle color changes earlier than human visual interpretation, reflecting its sensitivity to small distributional shifts in hue values. While this suggests potential for earlier detection, such use would require careful calibration of decision thresholds (e.g., *TH*) in conjunction with optimization of reaction chemistry and incubation protocols. Operationally, calibration would consist of (i) running a small chemistry-matched titration experiment that defines the minimum hue-distance shift corresponding to a true early positive, (ii) selecting *TH* such that the algorithm’s positive call coincides with that shift on the calibration data, and (iii) cross-validating against a fluorescence or qPCR reference on the same samples to ensure that algorithm-positive at the early time point corresponds to a real biochemical event and not to noise. We therefore consider early positive detection as a promising direction for future work.

### 5.2 Sensitivity Analysis on *TH*

The threshold coefficient *TH* was selected empirically, as it jointly governs the strictness of control validation and color discrimination. Given its importance, we performed a sensitivity analysis across all three data sets to evaluate the robustness of this parameter, the results of which are illustrated in Figure S6.

On paired tube-level calls from the *TH* sweep (same tubes evaluated at TH = 0.2 and TH = 0.3), McNemar’s test is nonsignificant at the 5% level on all three data sets, confirming that the two end-points of the plateau cannot be distinguished statistically; TH = 0.3 is therefore a defensible choice within the plateau.

Across all data sets, both rejection accuracy and color-detection accuracy remained consistently high within a *TH* range of approximately 0.2–0.3, despite data set-specific differences in the detailed response curves. This consistent stable region across three distinct experimental settings supports the robustness of the chosen threshold and indicates that the reported performance is not the result of fine-tuning to a single data set.

For deployment in new environments with substantially different imaging devices or lighting conditions, *TH* can be calibrated using a small pilot data set following the same analysis procedure. Our recommendation is a pilot of 30–50 panels covering both control-valid and known control-invalid cases. This is large enough to localize the plateau shown in Figure S6 but still far below the effort required for model training. A precise minimum will depend on the specific deployment noise profile and is identified as a deployment-phase validation question. This further highlights the flexibility and generalizability of the proposed relative distance-based framework, without reliance on a fixed, absolute threshold. The *TH*-vs-accuracy curves in Figure S6 are characteristically plateau-shaped: rejection accuracy rises steeply at small *TH* and enters a plateau in the approximate range TH ∈ [0.2, 0.3], in which both rejection and color-classification accuracy remain close to their peak, then degrade smoothly at larger *TH*. The plateau is the recommended operating region for any new deployment. Outside the plateau the degradation is gradual. As *TH* is reduced below the plateau, the rejection accuracy decreases due to over-rejection of borderline-valid panels, and as *TH* is increased above the plateau, the accuracy decreases due to under-rejection of borderline-invalid controls, with no abrupt cliff in the explored range 0.05 ≤ *TH* ≤ 0.50 (Figure S6).

### 5.3 Consequences of Misclassification on the Workflow

The “Half-empty” issue in Section 4.3 occurs due to human errors or delayed panel storage. Delays between running the test and capturing the result may cause shifted color hue, and user handling errors may lead to reduced solution volume. Even under this extreme circumstance of “half-empty”, the proposed workflow can still correctly identify the target status, which suggests practical robustness in challenging use scenarios.

Specific abnormalities that increase the rejection ratio include operator errors (panel misplacement producing cropping mismatch, large air bubbles obscuring the reaction volume) and extreme lighting (strong direct sunlight causing specular highlights, or insufficient ambient light casting shadows that blur tube boundaries). However, they will not influence the final detection accuracy, as these panels are rejected during tube check and excluded from further steps. From a deployment-usability perspective, the nominal false-rejection rate translates to a small fraction of tests requiring a retake per unit of throughput; in realistic settings, most of these are recoverable by a fresh photograph of the same panel without consuming a new panel, so the operational cost in terms of panel waste is materially lower than the headline false-rejection rate. After integrating the proposed algorithm flow in the Dragonfly App, when the tube check fails, user alerts could provide instructions such as adjusting the camera angle, retaking photos, or replacing the panels. The efficacy of such in-app alerts has not been prospectively measured in this study; our current interpretation is therefore qualitative only. Post hoc review of the false rejections suggests that cropping mismatch and extreme lighting were the most commonly observed failure modes, so guided photography should plausibly reduce at least part of the current rejection burden. Future efforts will focus on enhancing the photo cropping process to handle various panel placements and conducting a controlled comparison of guided vs unguided photography.

### 5.4 Importance of Using Relative Color-Distancing

Although the *H* value is considered robust against lighting changes, variability in the production (e.g., lyophilization process) or formulation (e.g., different batches of reagents) may still affect the color of positive/negative reactions, resulting in different *H* values among batches and among reactions. Small shifts in the absolute hue of positive/negative reactions can plausibly arise across production batches or assay runs; the relative-distance metric is designed to normalize the decision to the in-panel reference pair (Tubes 1 and 7–8), not to an absolute hue threshold. This is the design reason for using a relative, not absolute, color metric; a dedicated batch-to-batch quantification study lies outside the scope of the present manuscript. We envision vast applications of the proposed workflow, with its adaptive and relative decision strategy, in future scenarios of POC cLAMP diagnosis without the need for sophisticated adjustment, as demonstrated when applied to Data set 3.

Thanks to its adaptive and training-free framework, the proposed algorithm flow, initially developed for cLAMP-based applications, can also be generalized for other colorimetric amplification chemistries such as colorimetric rolling circle amplification (RCA) and strand displacement amplification^47^ as long as the contrasting colors of positive and negative references are provided. Generalization to a new chemistry has not yet been demonstrated in this work. It would require (i) introducing tube positions that carry the chemistry-appropriate positive and negative reference colors, (ii) rederiving the per-tube active-pixel threshold from the physical tube geometry of the new panel layout, and (iii) a small pilot data set to locate the *TH* plateau for the new color pair.

By using a statistical distance metric for color comparison, each processing step is explicit, deterministic, and grounded in physical or chemical rationale, and intermediate outputs can be inspected for validation and troubleshooting. The use of fixed, nonlearned decision criteria reduces the risk of model drift and facilitates traceability, which are key considerations for regulatory approval of medical device software. This closes the loop on the interpretability and regulatory concern raised in the Introduction: by confining the data-driven component to a locked, inference-only segmentation mask and placing every clinically consequential decision inside a deterministic, relative-distance rule set, the workflow is designed to meet exactly the traceability and model-drift requirements that end-to-end learned classifiers typically struggle with.

### 5.5 Adaptive and Training-Free Strategy

The workflow is characterized here as “training-free” and “adaptive” in two specific and restricted senses. “Training-free” means that no gradient-based optimization is performed against any labeled subset of the evaluation data: the deep segmentation component is used off-the-shelf, and the empirical hyperparameters (Canny thresholds, 14,000-pixel count, 20% contamination ratio, *TH*) are fixed defaults justified by physical geometry or literature practice, not by fitting to this work’s accuracy labels.

The term “adaptive” refers to the use of relative and context-aware decision strategies. Specifically, color identification is performed by comparing distributions within each panel using relative distance metrics (e.g., MMD distance), not absolute thresholds, allowing the algorithm to accommodate batch-to-batch variation, lighting changes, and formulation differences without manual recalibration. Adaptivity is therefore achieved through relative comparison and rule-based adjustment, not through data-driven learning. While *TH* itself is a fixed coefficient across the three data sets reported here, the adaptivity is provided per-panel, not per-data set. Each photograph carries its own references (Tubes 1 and 7–8) and the decision rule adapts to the reference pair of the specific image, allowing it to compensate for batch and lighting differences at the point of use. Other parameters used in this approach, such as the Canny thresholds, the 14,000-pixel count and the 20% Isolation-Forest contamination, are all empirically chosen values. None of these parameters is optimized via gradient descent against a labeled subset of the evaluation data; they are fixed defaults justified by physical geometry or by practice in the cited literature, and the sensitivity analyses confirm that the reported accuracies are not sitting on tight parameter boundaries. This represents a meaningful distinction from data-driven training and justifies that our approach is training-free. As an example for the case of *TH*, we explored data set-specific calibration: in each of the three data sets, the per-data set optimum lies inside the shared plateau and only marginally improved performance over the shared *TH* = 0.3.

### 5.6 Ablation Studies

To assess the necessity of individual components, ablation studies were conducted by bypassing pixel cleaning and fine segmentation. As shown in Table S1, bypassing pixel cleaning has little impact on color detection accuracy but reduces rejection accuracy in Data set 1. Bypassing both fine segmentation and pixel cleaning leads to a substantial degradation in rejection performance, particularly for Data set 3 collected under uncontrolled field conditions.

Although pixel cleaning provides limited benefit under typical conditions, it plays an important role in handling edge cases at negligible additional computation cost relative to the TRACER inference step. Under extreme or highly nonuniform lighting, pixel cleaning more effectively suppresses artifact regions that can bias color statistics. Such cases are unavoidable in real-world point-of-care deployment; therefore, pixel cleaning is retained as a key design choice to prioritize robustness and fail-safe behavior. Although the full-pipeline vs pixel-cleaning-bypassed difference in headline color-classification accuracy is small, the in-tube hue distribution is visibly broader without cleaning (see Figure 3a), which increases the probability of spurious *Unknown* calls on near-threshold tubes under field lighting; retaining pixel cleaning therefore protects the robustness of the rule, not its point accuracy. Table S1 contrasts the full pipeline against (a) bypassing pixel cleaning only and (b) bypassing both fine segmentation and pixel cleaning. While we do not claim a fully decomposed attribution between the two stages, the comparison supports the qualitative conclusion that the two stages together account for the rejection-accuracy gap on uncontrolled field data, with isolated attribution identified as future work.

### 5.7 Limitations and Future Work

While expert-adjudicated visual readouts were used as the reference in this study, the proposed workflow is specifically designed to operate in real-world POC settings where such expert review is unavailable and to remove reliance on user interpretation, thereby enabling standardized and reliable automated interpretation. This is not a contradiction: expert review was used once, at validation time, to establish the ground-truth labels that every machine-readable evaluation must be anchored against; the deployed workflow does not require expert input at any point. In the field-validation data set (Data set 3), the experts adjudicated the returned photographs offline and were never present at the participant’s side; the algorithm therefore has to function and is evaluated, in the exact same conditions it is deployed in.

The analytical and clinical performance of the Dragonfly assays themselves, including comparisons against molecular reference methods, have been validated and reported in our previous publications,^36,37^ demonstrating over 94% of sensitivity and 96% of specificity across various pathogens. Those prior figures refer to the assay as read manually; we emphasize that the present work measures algorithm-vs-manual agreement, not a head-to-head clinical comparison between algorithm and gold-standard reference on clinical samples. A prospective head-to-head study, in which both a human reader and the algorithm are evaluated on the same clinical samples with a molecular reference (qPCR), is identified as the key future-work item to close this gap.

The algorithm flow is intended to be embedded in a user app on portable devices, raising concerns about computational efficiency. The reported processing time of 1.05 s per sample was measured on a workstation under batch-processing conditions and does not directly represent device-level execution. However, the workflow is fully unsupervised and inference-only, avoiding computationally expensive model training.

For single-sample inference, performance gains on server hardware mainly arise from parallelism, not per-core acceleration. Modern mobile CPUs operating at higher clock frequencies have been shown to achieve comparable performance for on-device inference workloads.^48^ In addition, contemporary mobile system-on-chips increasingly incorporate dedicated neural processing units (NPUs) optimized for neural network inference, providing substantial speedups over general-purpose CPU or GPU execution for networks of comparable scale.^49^

The remaining steps of the pipeline are lightweight CPU-based operations, making deployment on tablets or smartphones feasible without orders-of-magnitude increases in processing time. This work is limited to evaluation on a workstation and a formal on-tablet benchmark has not been conducted; the deterministic image-processing stages are lightweight, and the TRACER inference is expected to dominate the per-sample time, in a regime that contemporary mobile hardware can plausibly support for single-photograph operation. On-device benchmarking is an explicit deployment-phase task (see the four-item program below). Future work will focus on device-level optimization, including network quantization and compression. Standard 8-bit quantization of comparable dense-vision networks is reported in the literature to deliver meaningful throughput improvement with limited accuracy loss^50^ and we explicitly flag maintenance of the headline accuracies as the go/no-go validation criterion for any deployed quantized variant, to enable a seamless photo-to-result experience in real-world point-of-care settings.

This design choice enables routine point-of-care operation to be performed fully on-device, without reliance on continuous Internet connectivity. Cloud-based processing is therefore intended as an optional mode for data aggregation, epidemiological reporting, and centralized analysis, not as a requirement for real-time diagnostic decision-making.

Although we tested the algorithm flow on thousands of reaction tubes taken under a variety of different lighting conditions, further validation in uncontrolled environments is necessary. Specifically, we identify the following deployment-scale validation program: (i) outdoor direct-sunlight and low-light evening conditions in the same field setting as Data set 3; (ii) multioperator studies in which each operator reads only the in-app feedback, not a trained expert’s guidance; (iii) a preregistered head-to-head comparison with a molecular reference (qPCR) on ∼500 clinical samples per panel; and (iv) prospective evaluation on photographs collected by color-vision-deficient operators to quantify any residual user-side bias. Future work will focus on integrating the proposed methods into the Dragonfly App and validating the entire workflow across a broader range of environments to demonstrate its compatibility with fully uncontrolled environments, leading toward implementation at the POC.

## 6 Conclusion

In this research, we proposed an automatic cLAMP readout workflow for multiple pathogen identification. The efficacy of the method was verified on 1,636 testing panel photos of 527 samples tested with the Dragonfly Kits, with variations of photo-taking devices and ambient lighting conditions. Evaluation results show over 96% accuracy in rejecting abnormal panel images with empty tubes or invalid controls, and over 99% accuracy in color detection. This multistep detection workflow, leveraging hybrid ROI segmentation and relative distance-based adaptive color identification, is fully automated and training-free and can be applied to other cLAMP testing scenarios without significant modifications, which is specified in the Discussion as deployment-time calibration only (placing positive/negative reference tubes in the new panel geometry, rederiving the geometric active-pixel threshold from the new tube dimensions, and locating the *TH* plateau on a ∼30-panel pilot, with no retraining). The proposed algorithm flow may be integrated with emerging cloud-based POC techniques; the deterministic image-processing stages and the MMD-based color decision can execute entirely on-device, so cloud processing is an optional mode for data aggregation, epidemiological reporting, and centralized analysis, not a requirement for the diagnostic decision. This also means no patient-linked image needs to leave the tablet for a routine readout, mitigating privacy concerns associated with cloud-based POC workflows while providing a powerful tool for the surveillance of infectious diseases and epidemiological investigations. Remaining limitations were identified in the Discussion, most importantly the absence of a prospective head-to-head comparison with a molecular reference, the remaining algorithm-vs-qPCR gap on clinical samples, the untested behavior under color-vision-deficient operators, and the ∼4.4% false-rejection rate. These define the scope of a program proposed in the Discussion for future deployment and validation of the platform.

## Supplementary Material



## Notes

Figure S1. The details of the Dragonfly Testing Kit; Figure S2. The Dragonfly sample-to-result workflow; Figure S3. Examples of three Dragonfly Testing Kits Involved; Figure S4. Algorithm-based detection results for the supplementary experiments, using *TH* = 0.3; Figure S5. Algorithm-based detection results for the supplementary experiments, using a stricter *TH* = 0.1; Figure S6. Sensitivity tests of *TH* vs rejection and color detection accuracy; Section S1. Experimental details and results of the supplementary experiments; Table S1. Ablation results of the algorithm flow (PDF)
