# Supplementary material for: AI-Driven Workflow for Automated Colorimetric Isothermal Amplification for Robust Pathogen Detection on Portable Devices
Source: ACS Omega. 2026 Aug 5;11(32):47285–301. doi: 10.1021/acsomega.5c12299 (PMC13491488; doi:10.1021/acsomega.5c12299)
Supplement: Supplementary file 1 [file ao5c12299_si_001.pdf]

AI-Driven Workflow for Automated Colorimetric Isothermal Amplification for Robust Pathogen Detection on Portable Devices

Ke Xu[a,b,c], Matthew L. Cavuto[a,b,c], Kenny Malpartida-Cardenas[a,b], Marcus Pond[d], Dimbintsoa Rakotomalala Robinson[e], Francois Kiemde[f], Bernard Hernandez[a,b,c], Shiranee Sriskandan[a], Umberto D'Alessandro[e], Annette Erhart[e], Halidou Tinto[f], Aubrey J. Cunnington[a], Alison Holmes[a,b,g], Pantelis Georgiou[c], Nicolas Moser[c,g,h,†], Jesus Rodriguez-Manzano[a,b,g,†,*]

[a]Department of Infectious Disease, Faculty of Medicine, Imperial College London, London SW7 2AZ, United Kingdom

[b]Centre for Antimicrobial Optimisation, Imperial College London, Room 7S5, Commonwealth Building, Hammersmith Hospital Campus, Du Cane Road, London W12 0NN, United Kingdom

[c]Department of Electrical and Electronic Engineering, Faculty of Engineering, Imperial College London, Exhibition Road, London SW7 2AZ, United Kingdom

[d]Department of Infection and Immunity, Imperial College Healthcare NHS Trust, St Mary's Hospital, Praed Street, London W2 1NY, United Kingdom

[e]MRC Unit The Gambia at the London School of Hygiene and Tropical Medicine, Atlantic Boulevard, Fajara, PO Box 273, Banjul, The Gambia

[f]Unité de Recherche Clinique de Nanoro, Institut de Recherche en Sciences de la Santé, BP 218 OUAGA 11, Nanoro, Boulkiemdé, Burkina Faso

[g]The Fleming Initiative, Imperial College London and Imperial College Healthcare NHS Trust, St Mary's Hospital, Praed Street, London W2 1NY, United Kingdom

[h]Department of Computing, Faculty of Engineering, Imperial College London, London SW7 2AZ, United Kingdom

†Both authors should be considered senior authors.

*Corresponding Author: j.rodriguez-manzano@imperial.ac.uk

Table of Contents

Figure S1. The details of the Dragonfly Testing Kit 2

Figure S2. The Dragonfly sample-to-result workflow 2

Figure S3. Examples of three Dragonfly testing kits involved 3

Figure S4. Algorithm-based detection results for the supplementary experiments, using TH=0.3 4

Figure S5. Algorithm-based detection results for the supplementary experiments, using a stricter TH=0.1 4

Figure S6. Sensitivity tests of *TH* vs. rejection and color detection accuracy 5

Section S1. Experimental details and results of the supplementary experiments 5

Table S1. Ablation results of the algorithm flow. 5

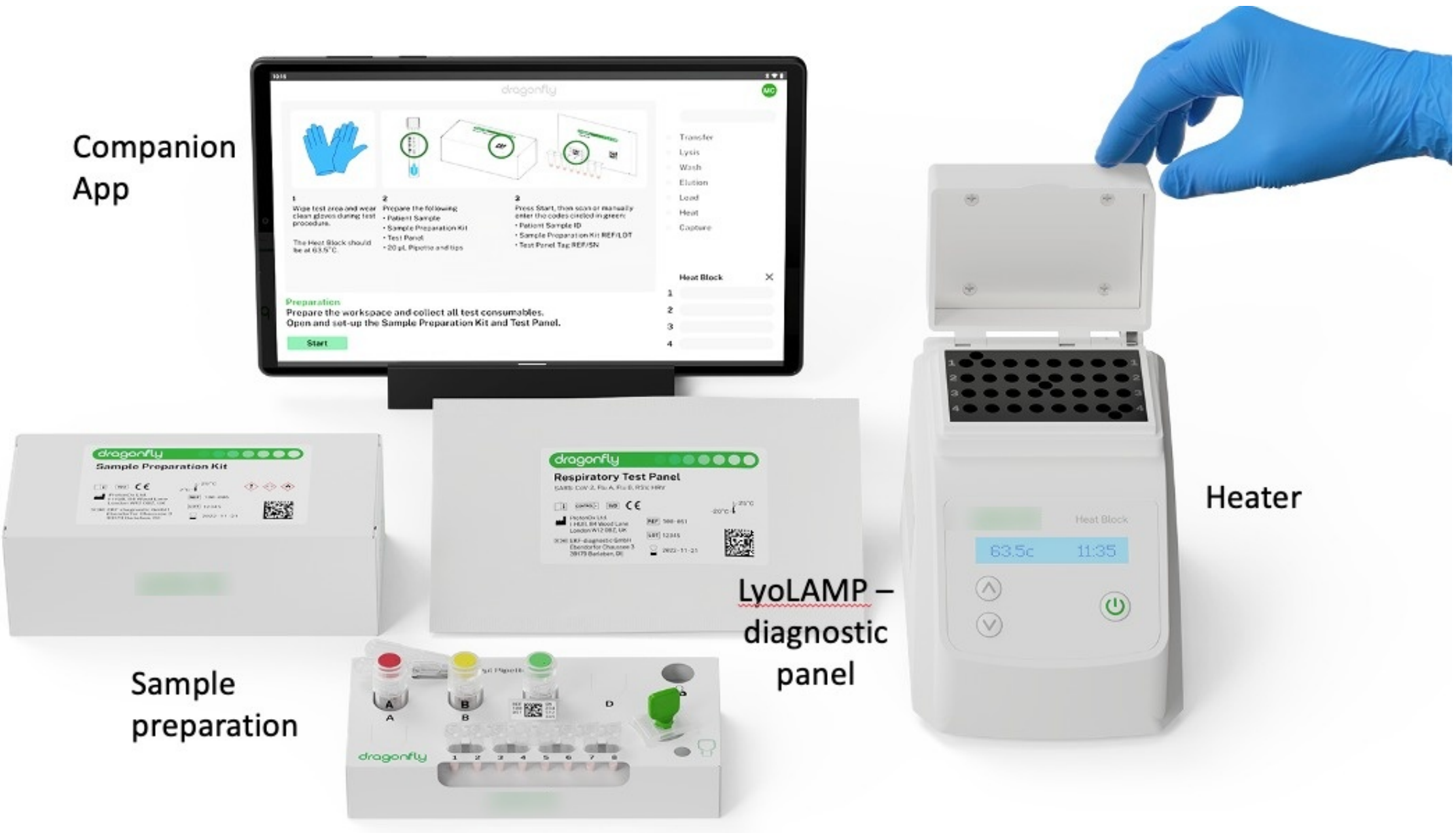

Figure S1. The details of the Dragonfly Testing Kit, which includes a sample preparation kit, a test panel (each panel tests five kinds of pathogens), a heating block, and an algorithm-integrated App running on tablets or smartphones.

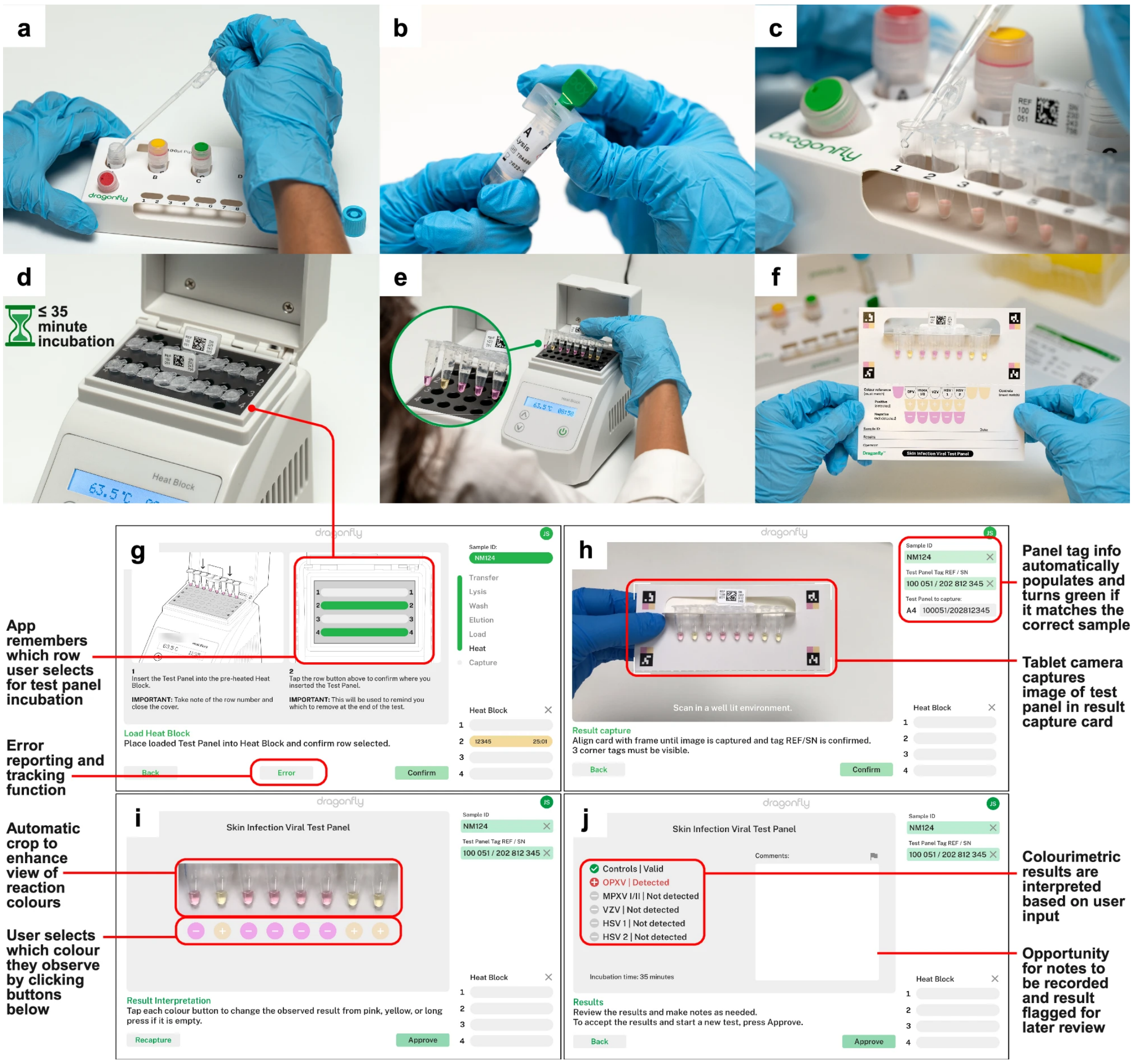

Figure S2. The Dragonfly sample-to-result workflow. Adapted from [36] under CC BY 4.0.

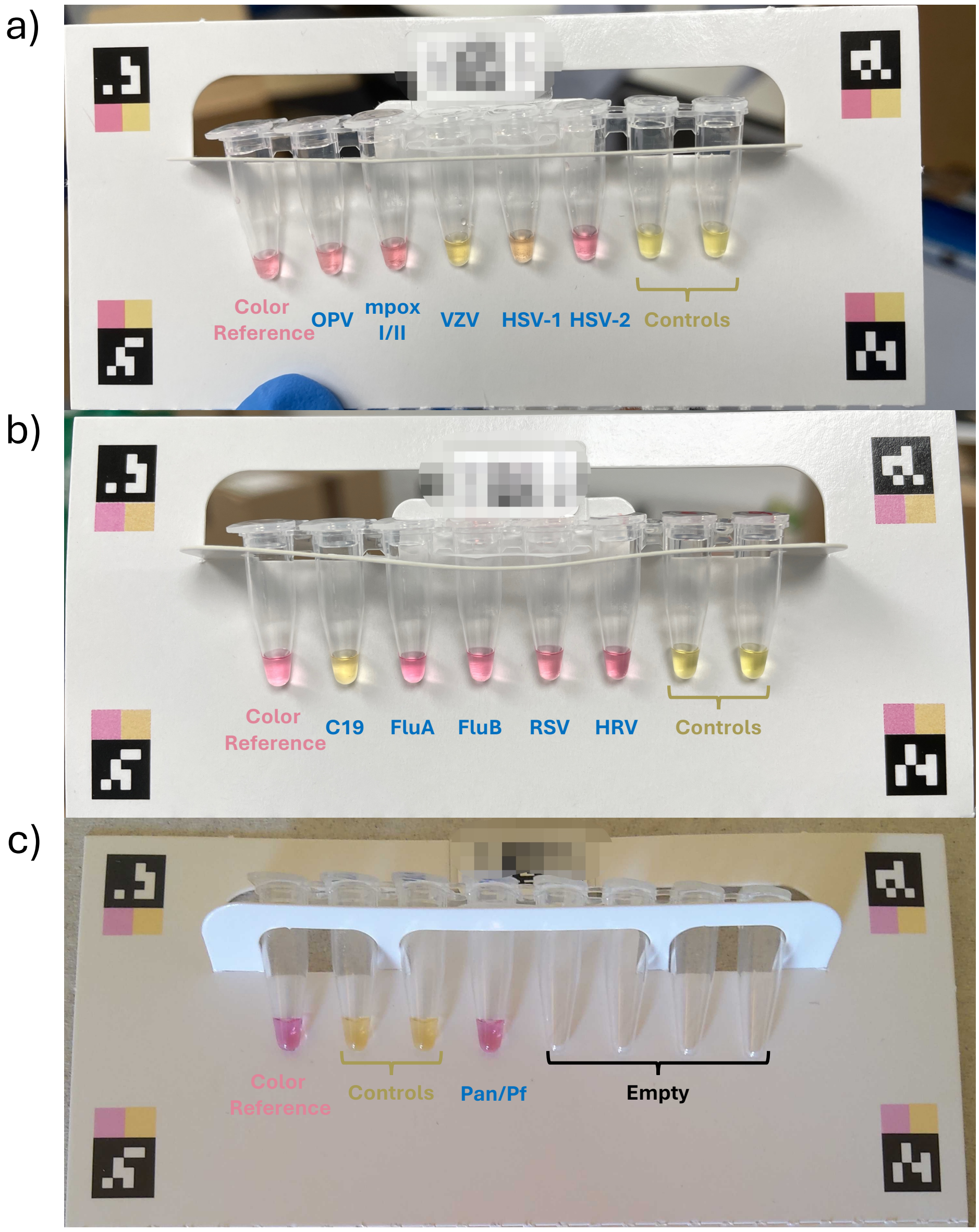

Figure S3. Examples of sample results from **a)** Skin Virus Panel, including orthopoxvirus (OPXV), monkeypox virus (mpox I/II), varicella zoster virus (VZV), human herpesvirus 1 (HSV-1), and human herpesvirus 2 (HSV-2); **b)** respiratory Panel, including SARS-CoV-2 (C19), influenza A virus (FluA), influenza B virus (FluB), respiratory syncytial virus (RSV), and human rhinovirus (HRV); and **c)** Malaria Panel for detection pan-malaria. For **a)** and **b)**, color reference and controls are distributed in the first and the last two positions, with Tube 2-6 as tests. c) Follows a different pattern in which color reference and controls lay in the first three positions, the test tube locates in the fourth, and the rest remain empty. Adaptations were carried out to apply the proposed algorithm flows on the Malaria Panel.

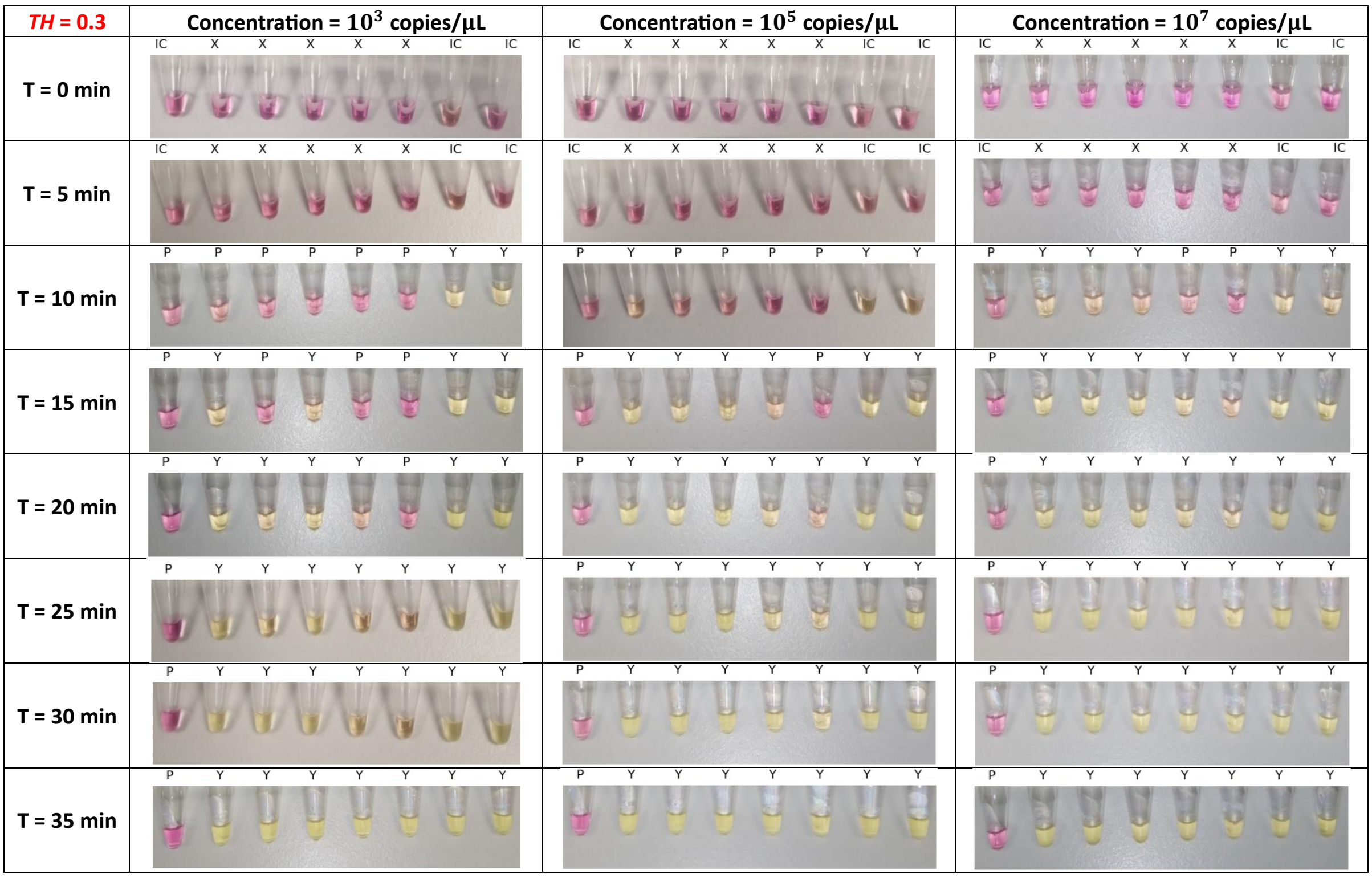

Figure S4. Algorithm-based detection results for the supplementary experiments, using *TH*=0.3. The detected color on top of each tube: P: pink/negative, Y: yellow/positive, U: unknown, IC: incorrect control, X: not relevant because of control check or empty check failure. T indicates the incubation time since started.

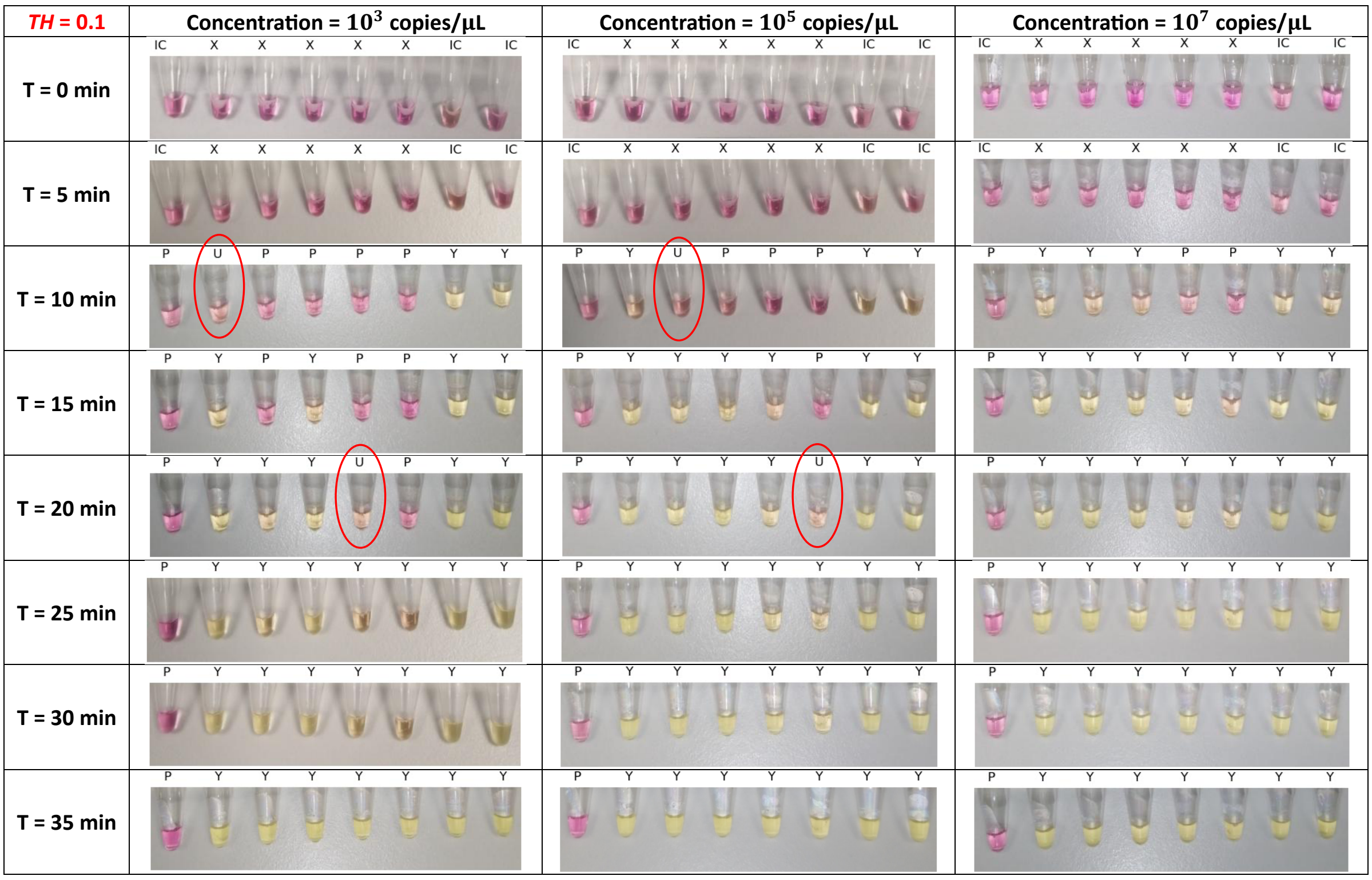

Figure S5. Algorithm-based detection results for the supplementary experiments, using a stricter *TH*=0.1. Tubes detected as 'Unknown' are circled as shown.

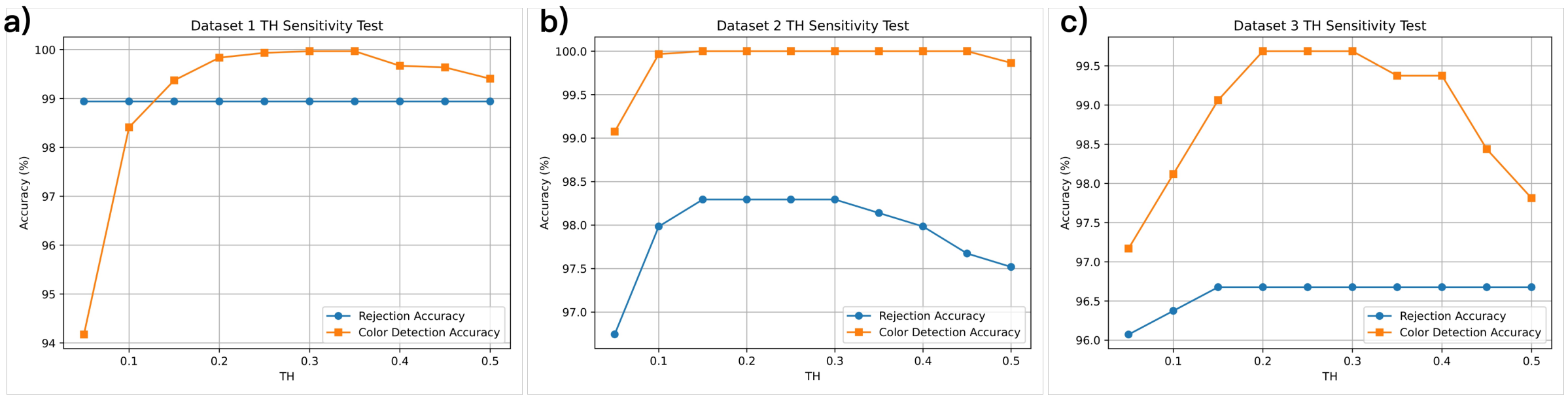

Figure S6. Sensitivity tests of TH vs. rejection and color detection accuracy for the three demonstrated datasets.

Section S1: Experimental details and results of the supplementary experiments

To explore the algorithm response to this abnormal situation and evaluate the method's sensitivity, we carried out supplementary experiments with Skin-tropic virus panels on synthetic DNA (gBlocks, IDT Inc., United States). Three concentrations (10^3, 10^5, 10^7 copies/μL) of all the five pathogen DNA were tested, and photos of the panels were taken at 0, 5, 10, 15, 20, 25, 30, 35 min after incubation started with a smartphone camera (13 MP, HUAWEI P smart 2019, China), to see the color transforming phases of tubes under various concentrations.

The proposed algorithm flow was applied to the photos taken in the supplementary experiments with the adjustable TH as 0.3 and 0.1 respectively, and the results are shown in Fig. S4 and Fig. S5 (for each tube, P: pink/negative, Y: yellow/positive, U: unknown, IC: incorrect control, X: not relevant because of control check failure). For all the concentrations, at 0 and 5 min no color changes (including the control tubes) can be observed as the reactions have just begun, and the algorithm correctly identified the abnormality in both figures. As the reactions went on, some tube colors started to transform due to the pH change in the solution, and ambiguous colors appeared for certain pathogens. Whether this transforming status can be captured or not depends on the target's Time-to-Positive (TTP) (which is related to the assay's chemical nature) and the initial concentration. When a moderate decision coefficient $TH = 0.3$ is set, positive reactions can be identified earlier as illustrated in Fig. S4, indicating the high sensitivity of detection for the algorithm. If stricter decisions are preferred with $TH = 0.1$ (as shown in Fig. S5), some hard-to-identify ambiguous colors will be considered 'in-between' and marked as 'Unknown' by the algorithm. In this case, user notifications will be pushed by the App to ask for longer incubation time. As long as the TH is set inside a reasonable range, the algorithm can perform robustly with high sensitivity.

Table S1. Ablation results of the algorithm flow.

Method	Dataset 1		Dataset 2		Dataset 3	
	Rejection Accuracy	Color Detection Accuracy	Rejection Accuracy	Color Detection Accuracy	Rejection Accuracy	Color Detection Accuracy
Full Algorithm	98.94%	99.97%	98.29%	100%	96.68%	99.69%
Bypassing Pixel Cleaning	98.33%	100%	98.29%	100%	96.98%	99.69%
Bypassing Fine Segmentation & Pixel Cleaning	96.52%	99.97%	97.52%	100%	81.87%	99.63%
